# Supplementary material for: Geometry design of tethered small-molecule acceptor enables highly stable and efficient polymer solar cells
Source: Nat Commun. 2023 May 22;14:2926. doi: 10.1038/s41467-023-38673-5 (PMC10203300; doi:10.1038/s41467-023-38673-5)
Supplement: Supplementary file 1 — Supplementary Information [file 41467_2023_38673_MOESM1_ESM.pdf]

# Supplementary Information

## Geometry Design of Tehered Small-molecular Acceptor Enables Polymer Solar Cells with Efficiency over 18% and High Stability

Bai, *et al.*

## Supplementary Figures

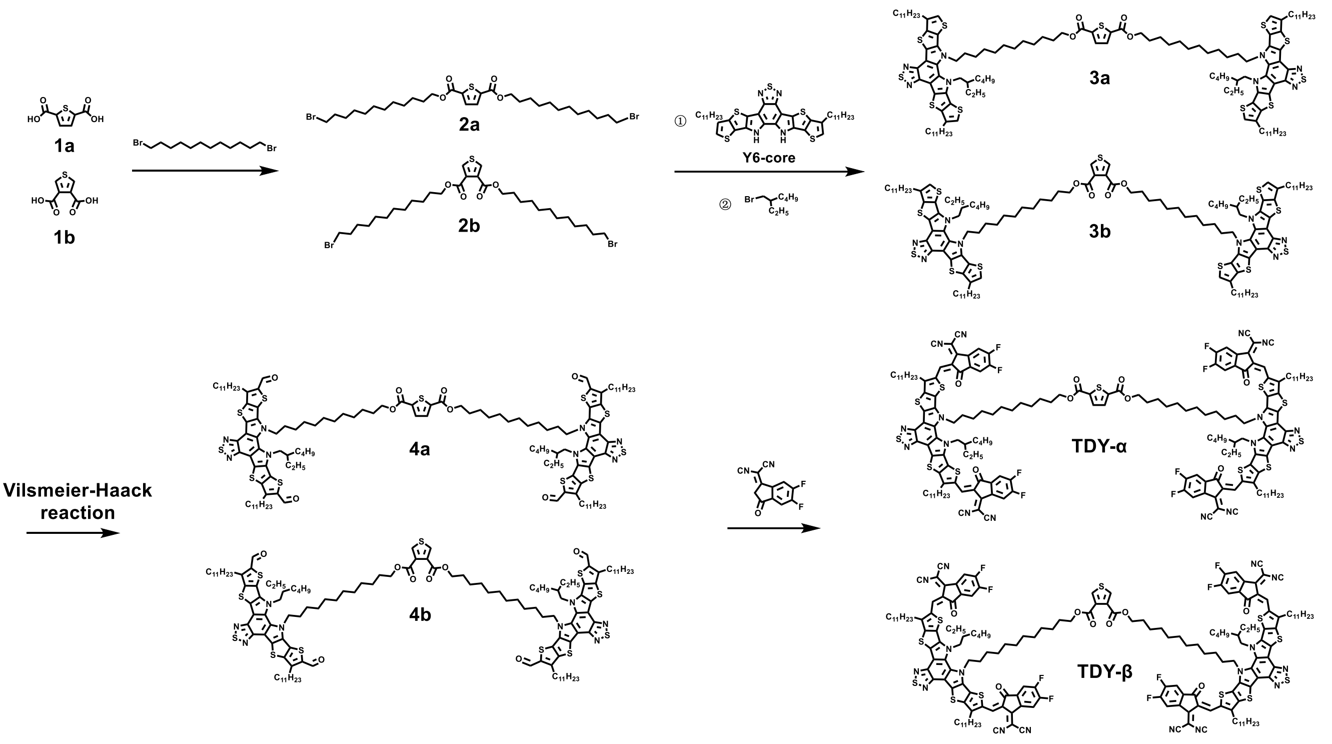


**Supplementary Figure 1.** Synthetic routes of TDY-α and TDY-β

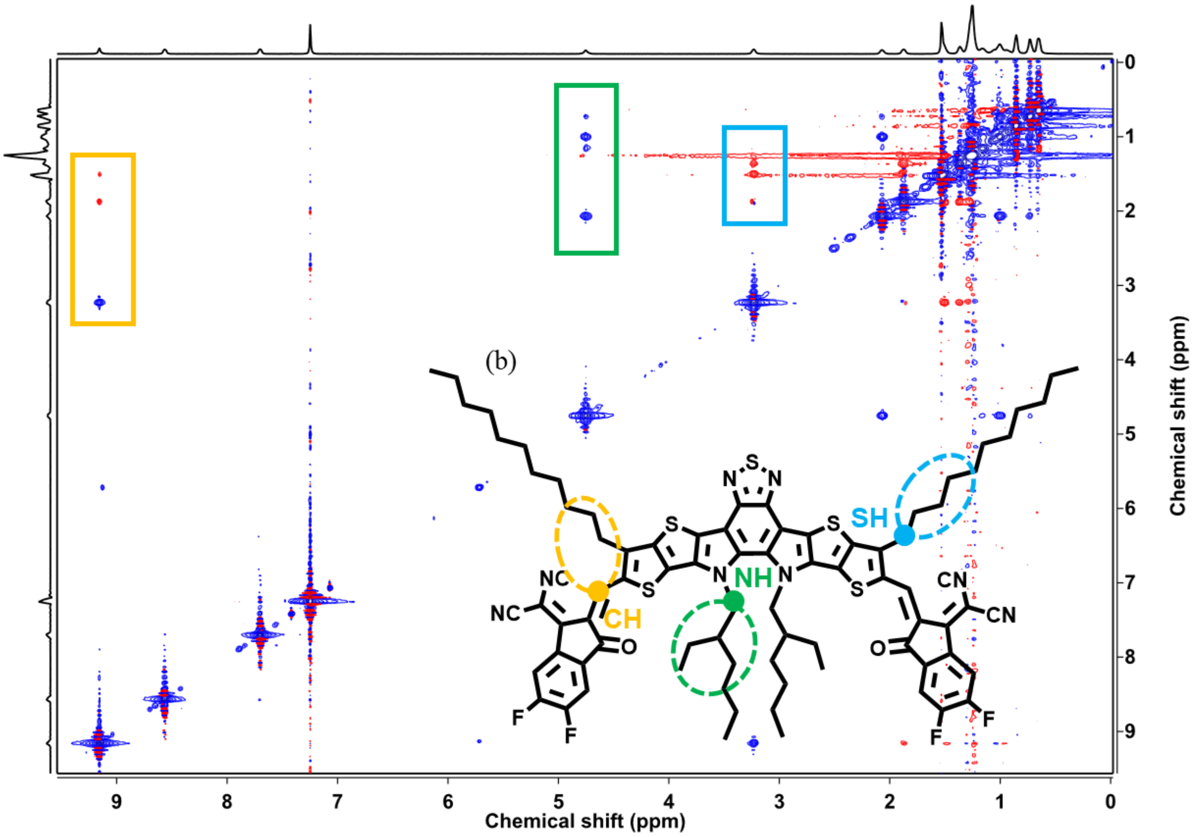


**Supplementary Figure 2.** $^1$H-$^1$H NOESY NMR spectra (328K, Full-scale) of Y6 with intramolecular interaction marks on the chemical structure of Y6. When a proton is saturated or inverted, spatially-close protons may experience an intensity enhancement, which is termed the Nuclear Overhauser Effect (NOE). The NOE does not depend upon through-bond J couplings but depends only on the spatial proximity between protons. For large molecules, an NOE signal may be observed between protons that are up to 5Å apart, and the strength of the NOE signal gives information on how close two protons are. The $^1$H-$^1$H NOESY NMR spectra of Y6 confirmed intrinsic intramolecular interactions in four regions, as the off-diagonal signals shown in Supplementary Figure 2:

1. among hydrogens from the aliphatic chains with nearby aliphatic hydrogen atoms (-$C_{11}H_{23}$ and -$C_8H_{17}$, upper right corner);
2. among hydrogens CH with nearby aliphatic hydrogen atoms on -$C_{11}H_{23}$ (yellow region);
3. among hydrogens NH with nearby aliphatic hydrogen atoms on -$C_8H_{17}$ (green region);
4. among hydrogens SH with nearby aliphatic hydrogen atoms on -$C_{11}H_{23}$ (blue region).

Beside these four intrinsic intramolecular interactions, there were no other obvious off-diagonal signals, which means that under the extreme-low concentration of testing samples, there were no obvious intermolecular interactions. Therefore, the off-diagonal signals presented in the $^1$H-$^1$H NOESY NMR spectra of TSMAs must be defined as the intramolecular interactions between two Y6 units on either side of the one TSMAs.

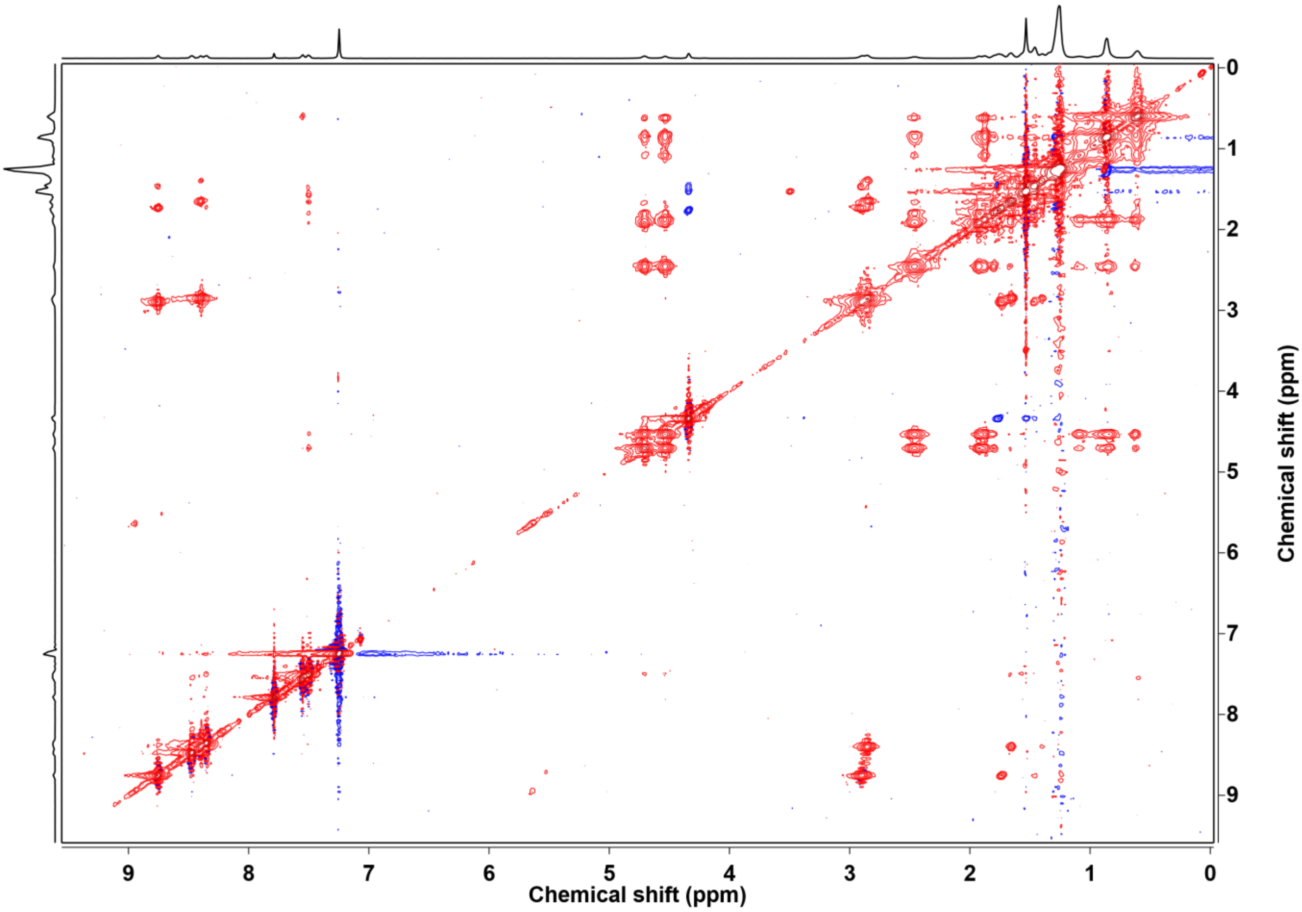


**Supplementary Figure 3.** $^{1}H$-$^{1}H$ NOESY NMR spectra (298K, Full-scale) of TDY-α

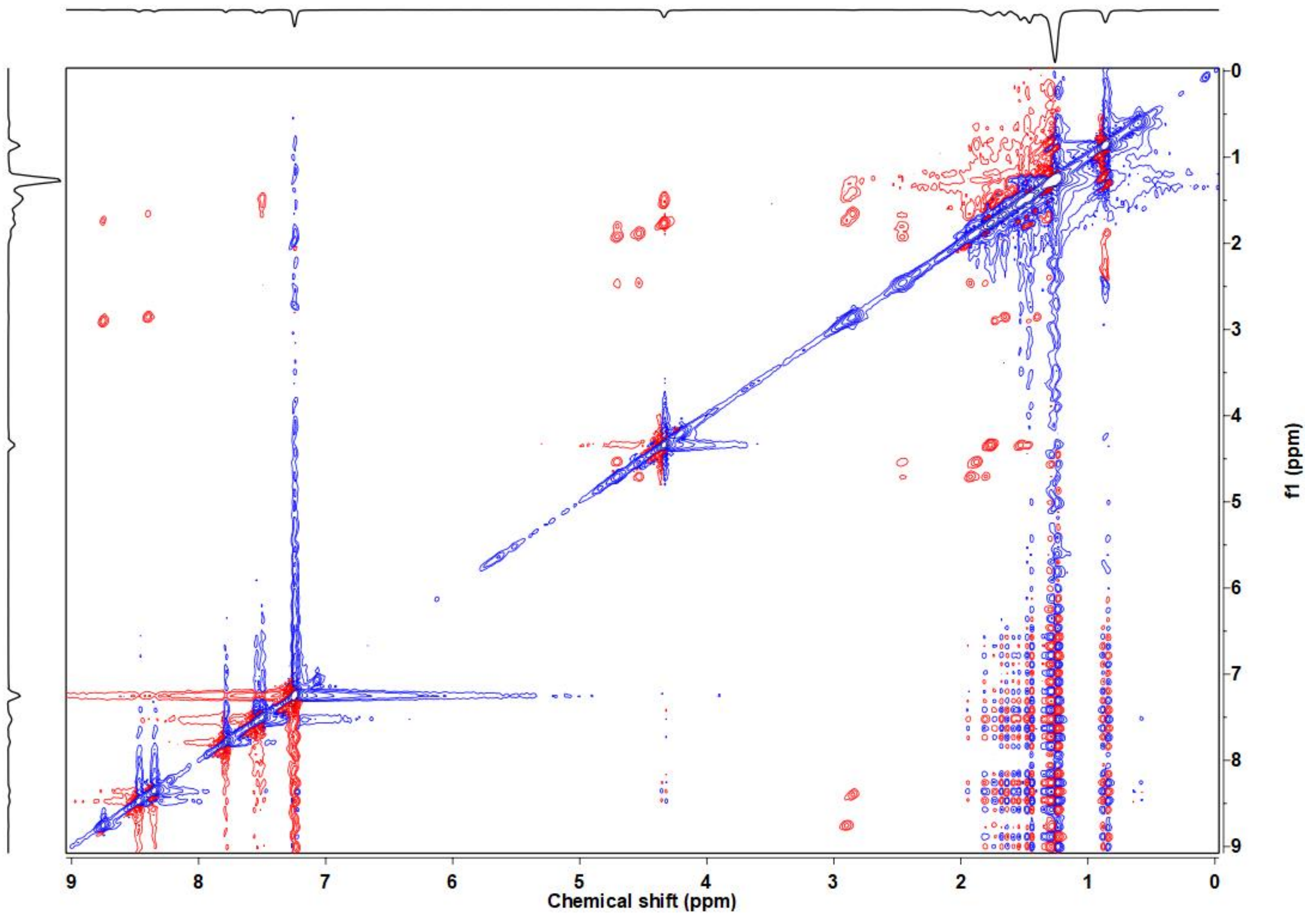


**Supplementary Figure 4.** $^{1}H$-$^{1}H$ ROESY NMR spectra (298K, Full-scale) of TDY-α

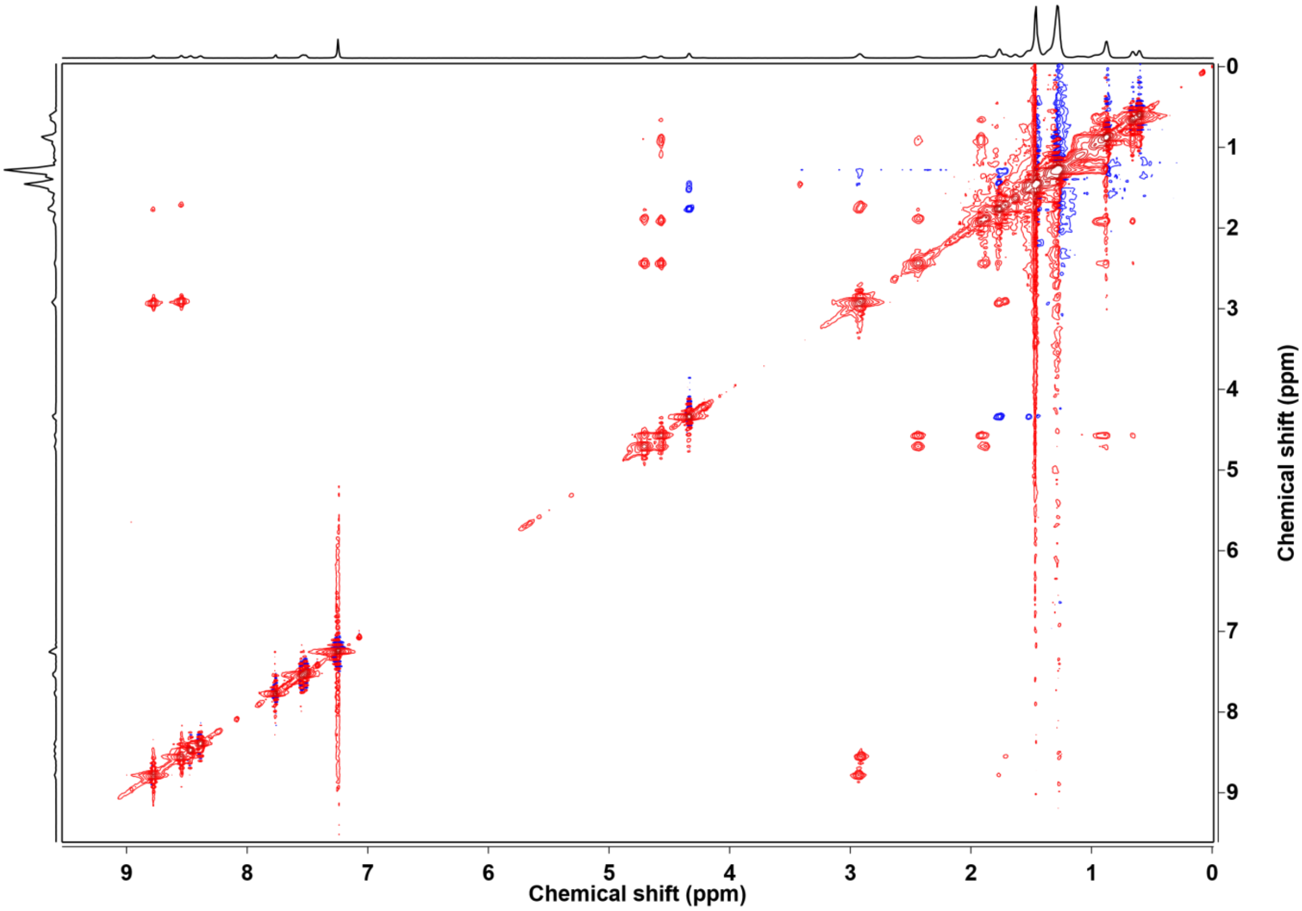


**Supplementary Figure 5.** $^1$H-$^1$H NOESY NMR spectra (328K, Full-scale) of TDY-α

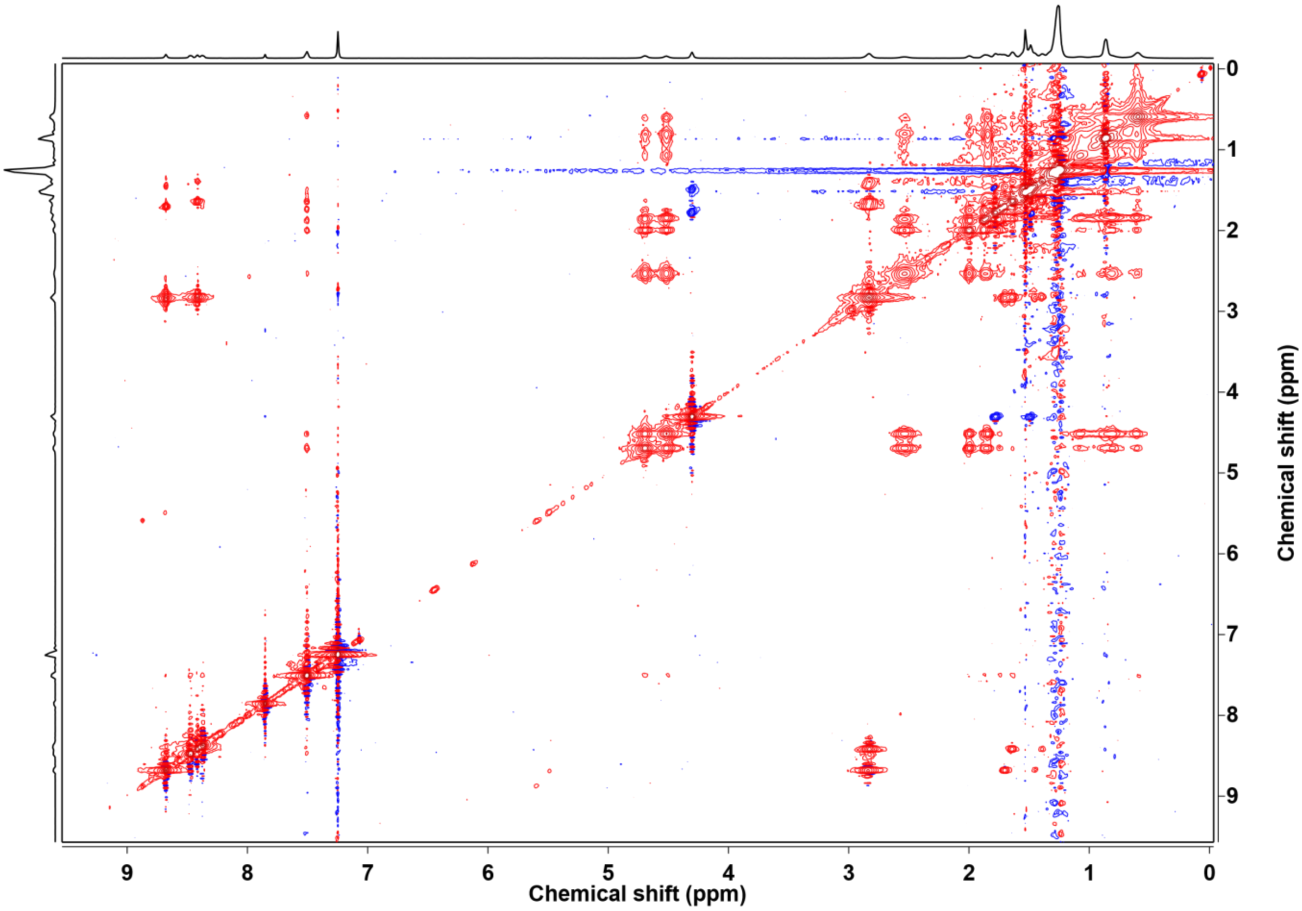


**Supplementary Figure 6.** $^{1}H$-$^{1}H$ NOESY NMR spectra (298K, Full-scale) of TDY-β

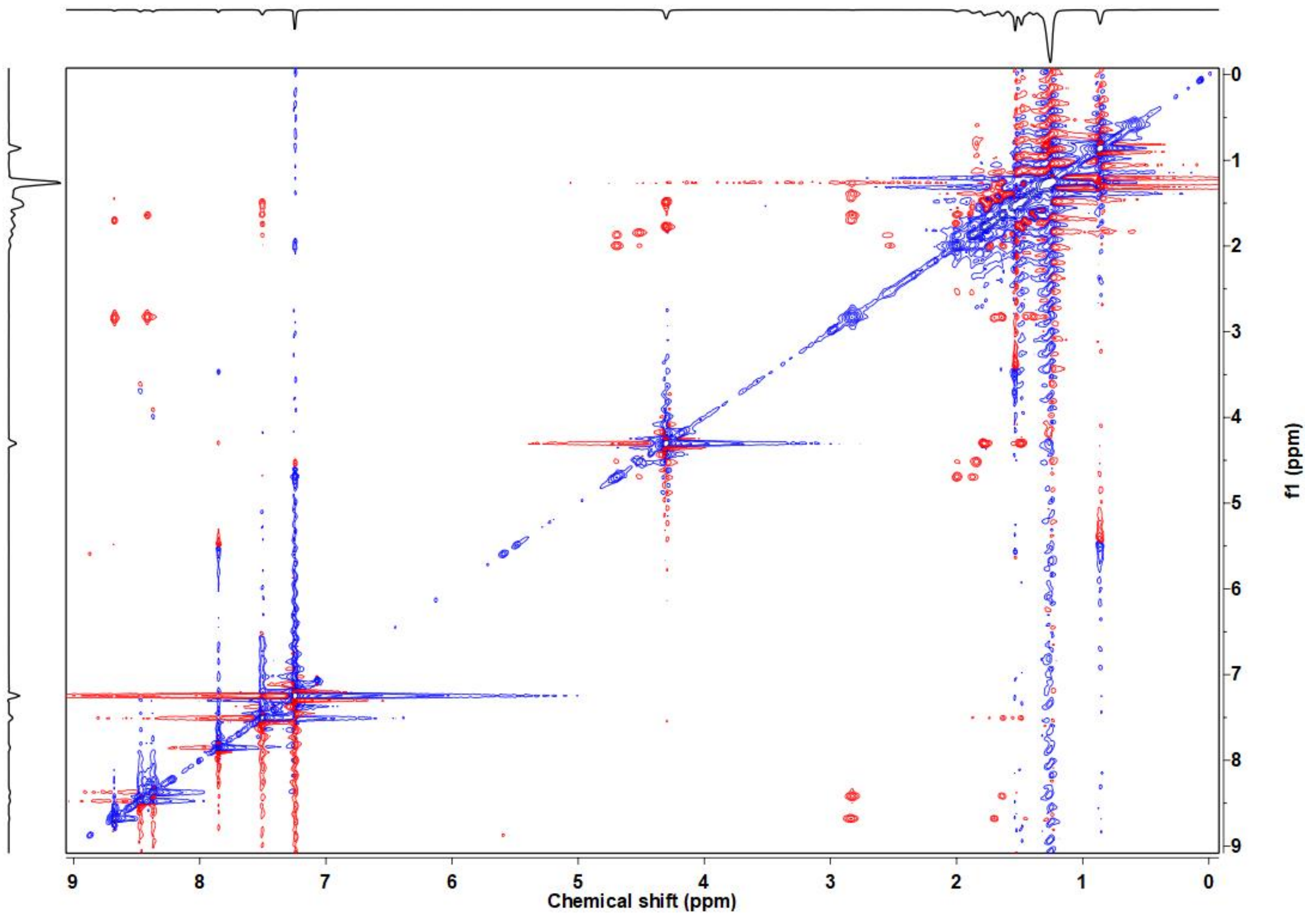


**Supplementary Figure 7.** $^{1}H$-$^{1}H$ ROESY NMR spectra (298K, Full-scale) of TDY-β

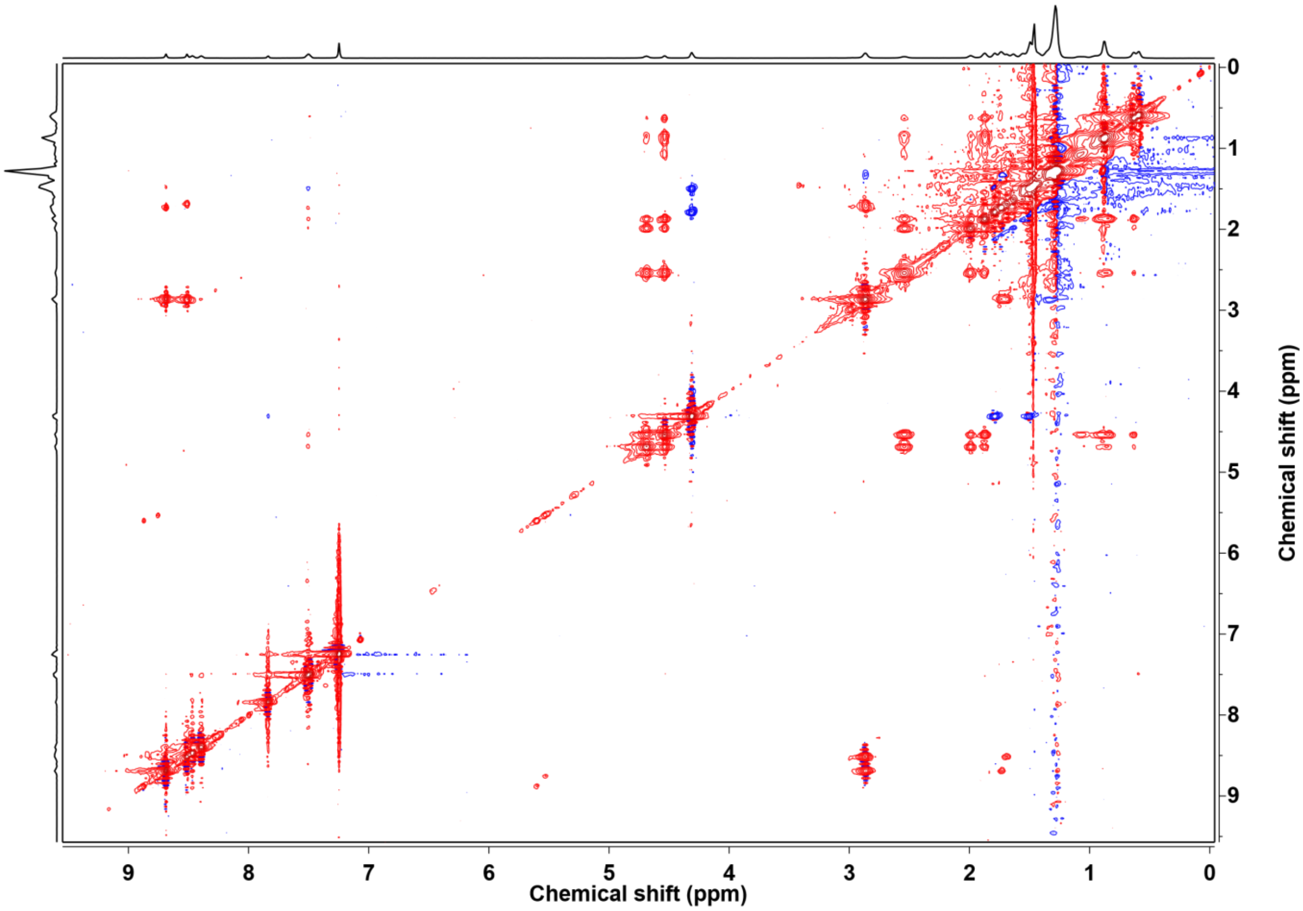


**Supplementary Figure 8.** $^{1}H$-$^{1}H$ NOESY NMR spectra (328K, Full-scale) of TDY-β

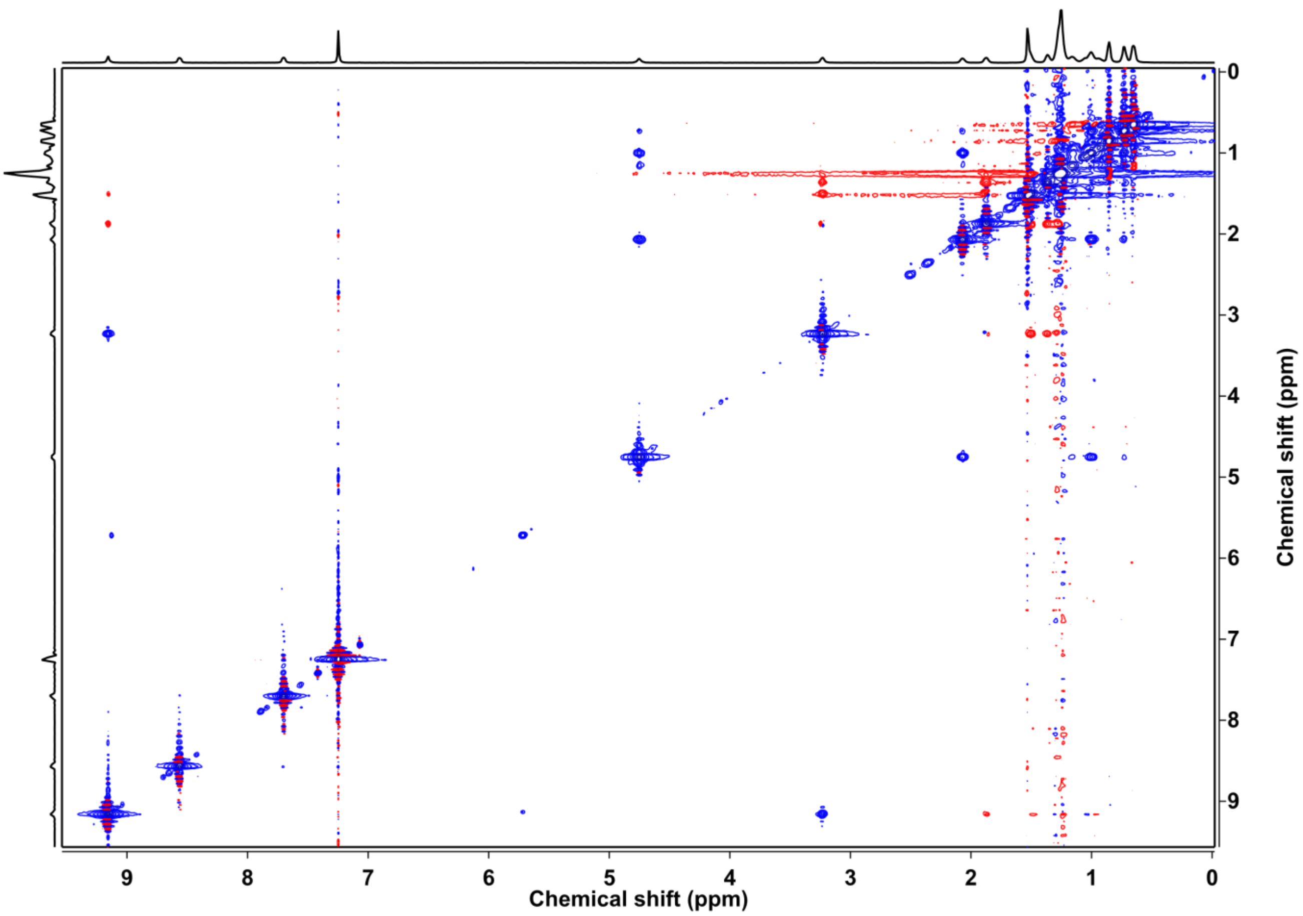


**Supplementary Figure 9.** $^1$H-$^1$H NOESY NMR spectra (298K, Full-scale) of Y6

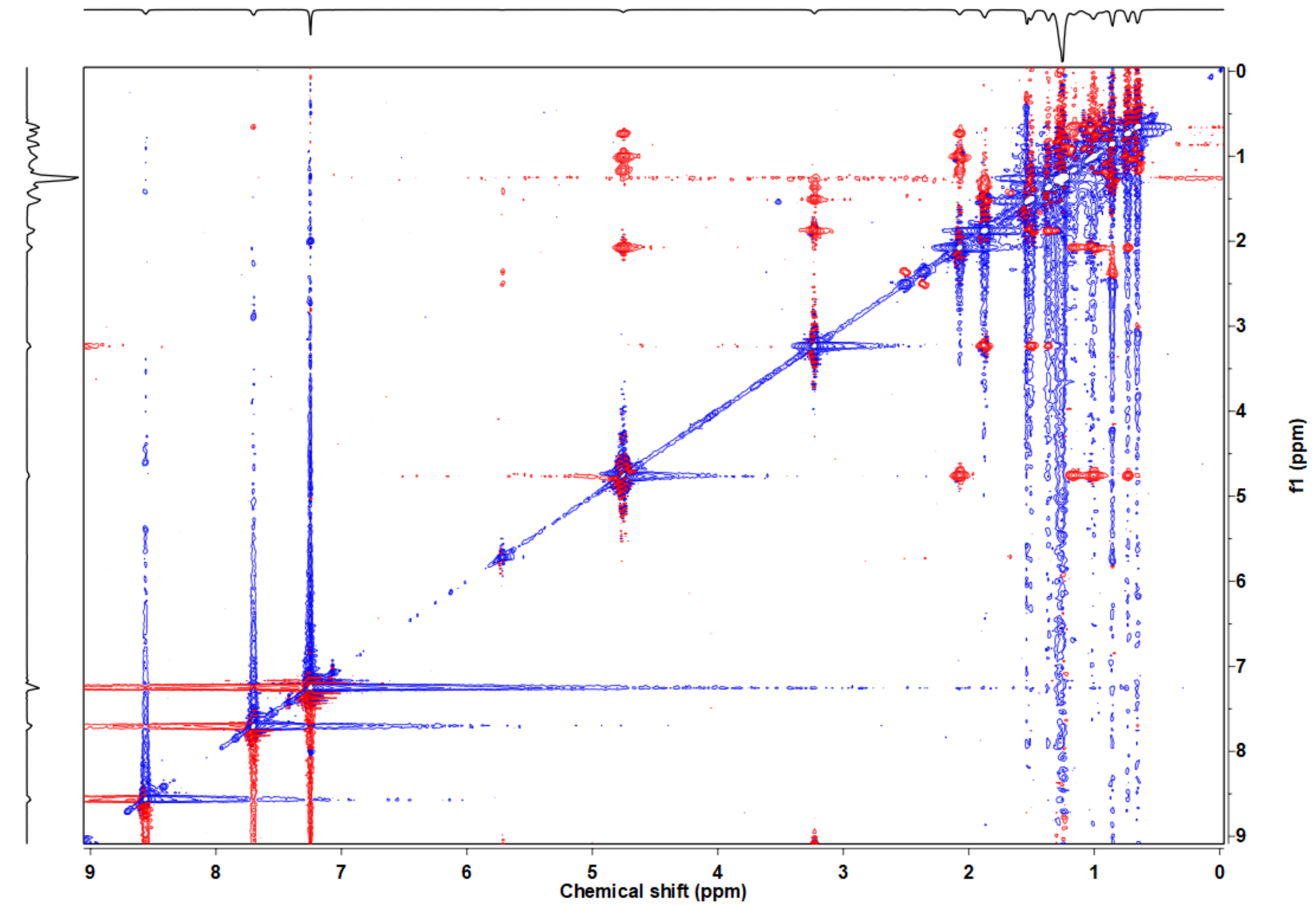


**Supplementary Figure 10.** $^{1}H$-$^{1}H$ ROESY NMR spectra (298K, Full-scale) of Y6

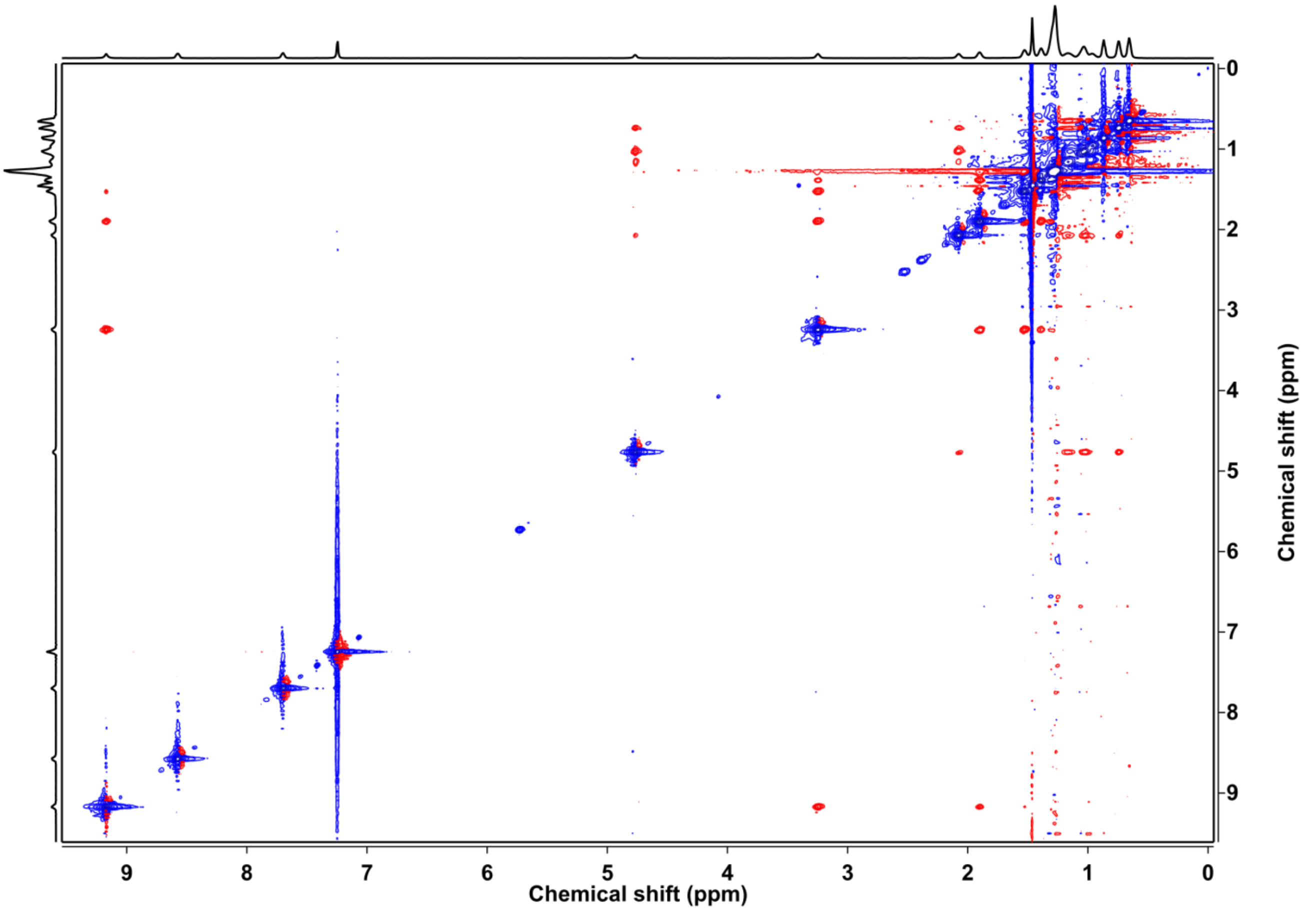


**Supplementary Figure 11.** $^1H$-$^1H$ NOESY NMR spectra (328K, Full-scale) of Y6

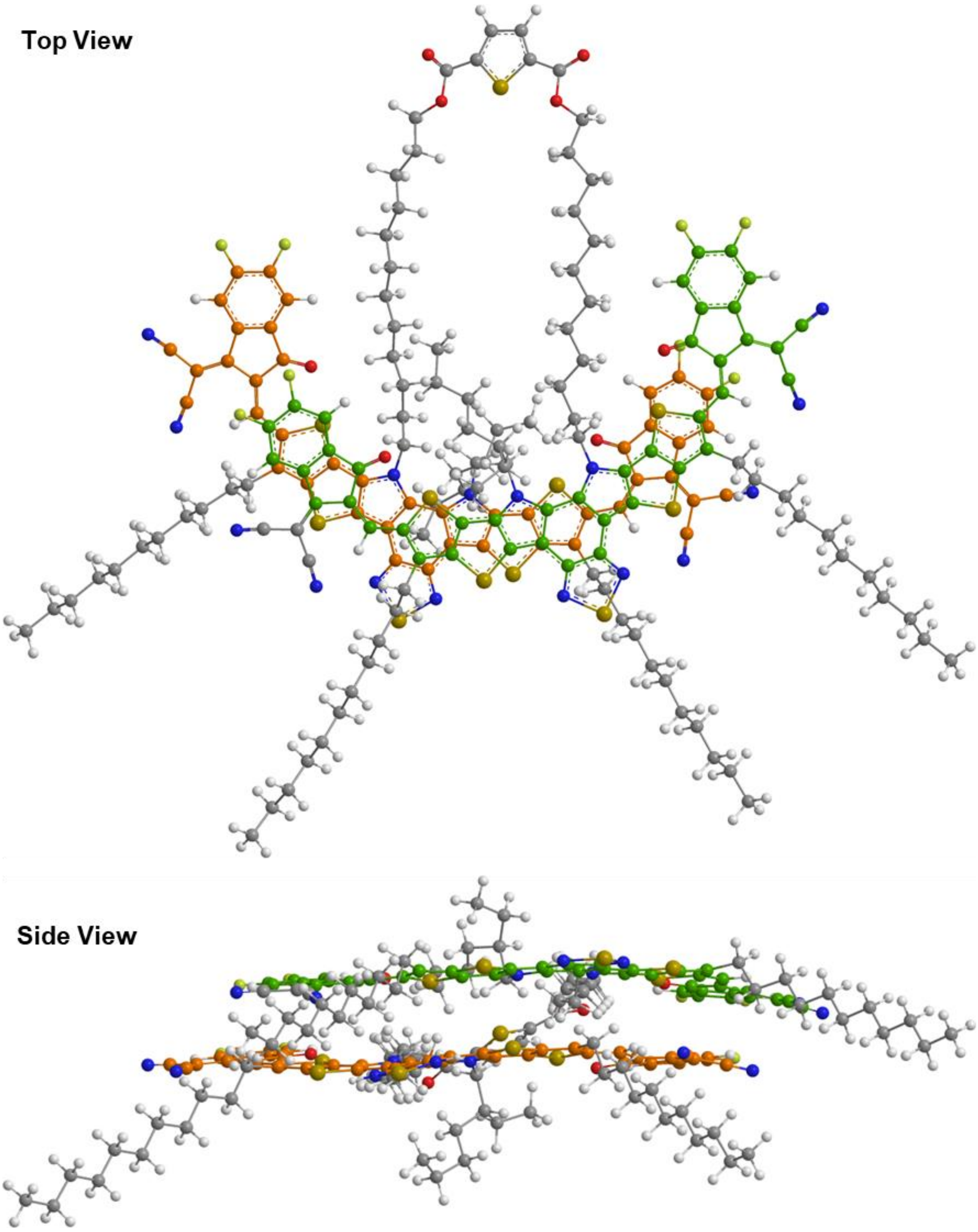


**Supplementary Figure 12.** The top view and side view of the optimal geometric configurations of TDY-α

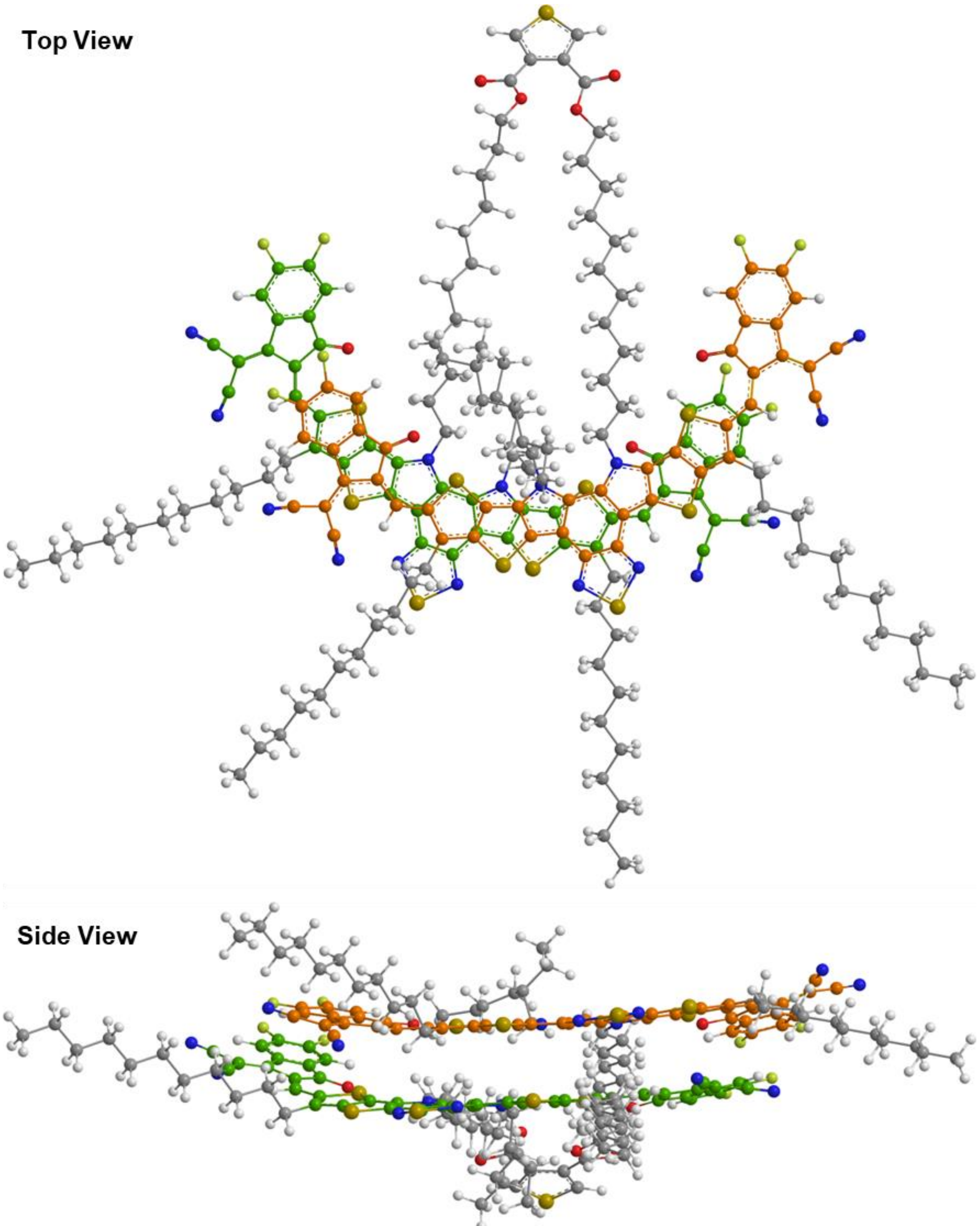


**Supplementary Figure 13.** The top view and side view of the optimal geometric configurations of TDY-β

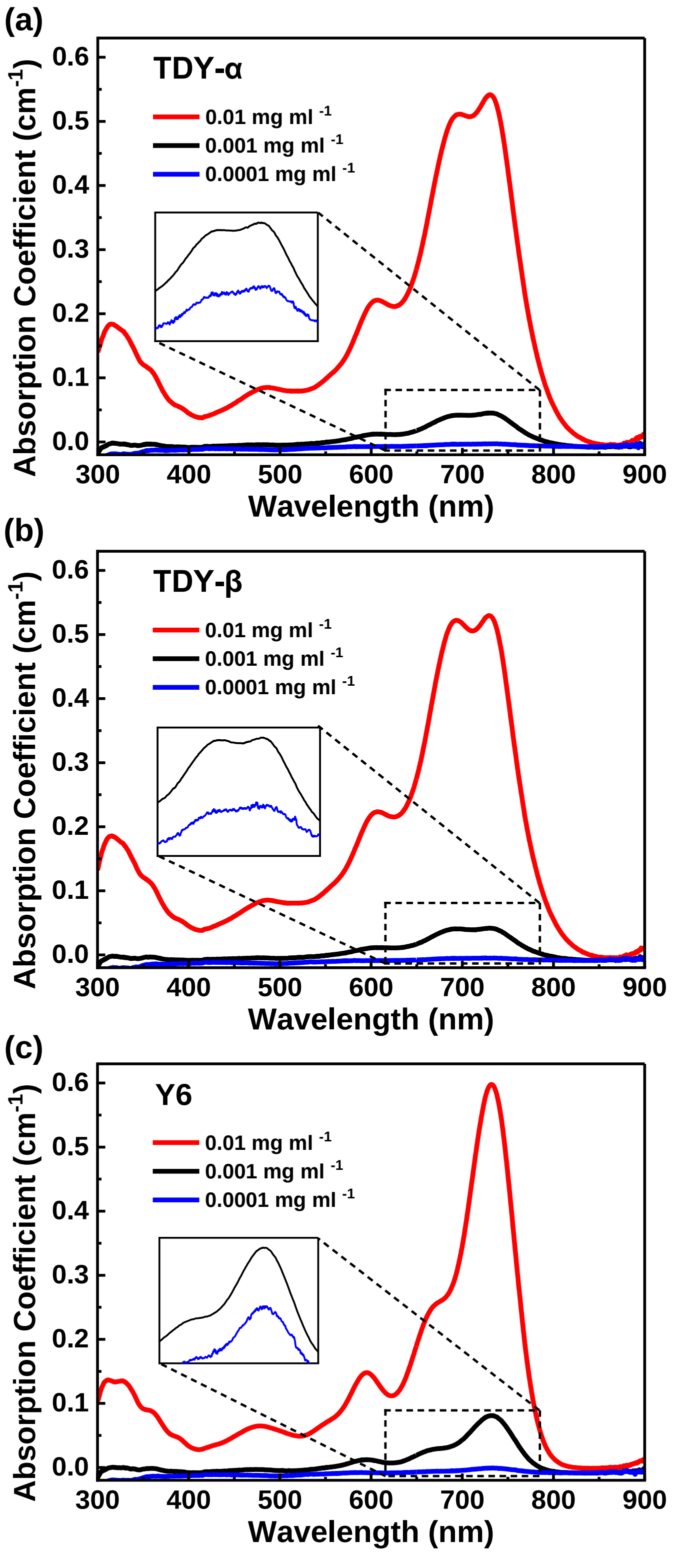


**Supplementary Figure 14.** Solution absorptions of the dimers with different concentrations. Normalized UV-vis absorption spectra of (a) TDY-α, (b) TDY-β and (c) Y6 in chloroform with various concentrations.

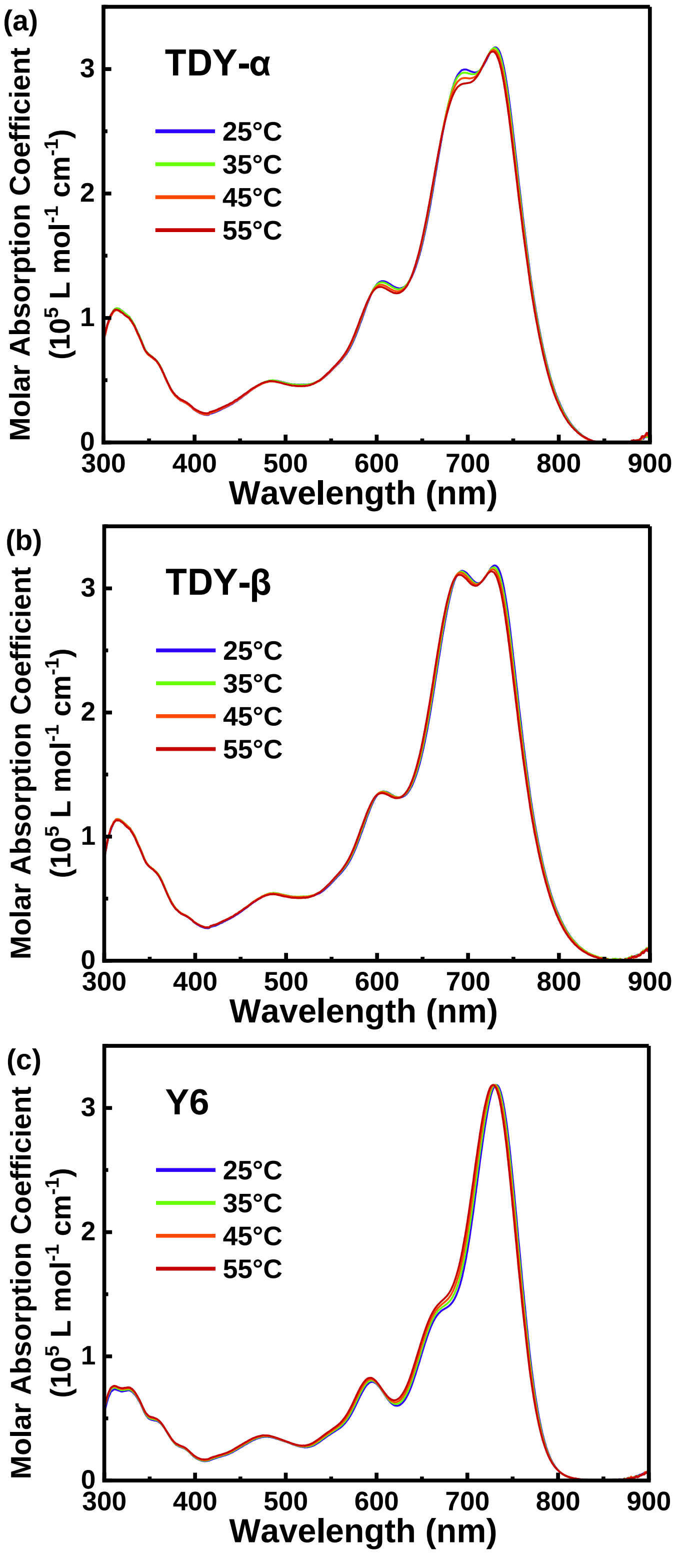


**Supplementary Figure 15.** Solution absorptions of the dimers with different temperature. UV-vis absorption spectra of (a) TDY-α, (b) TDY-β and (c) Y6 in chloroform (0.01 mg $ml^{-1}$) under increasing temperature.

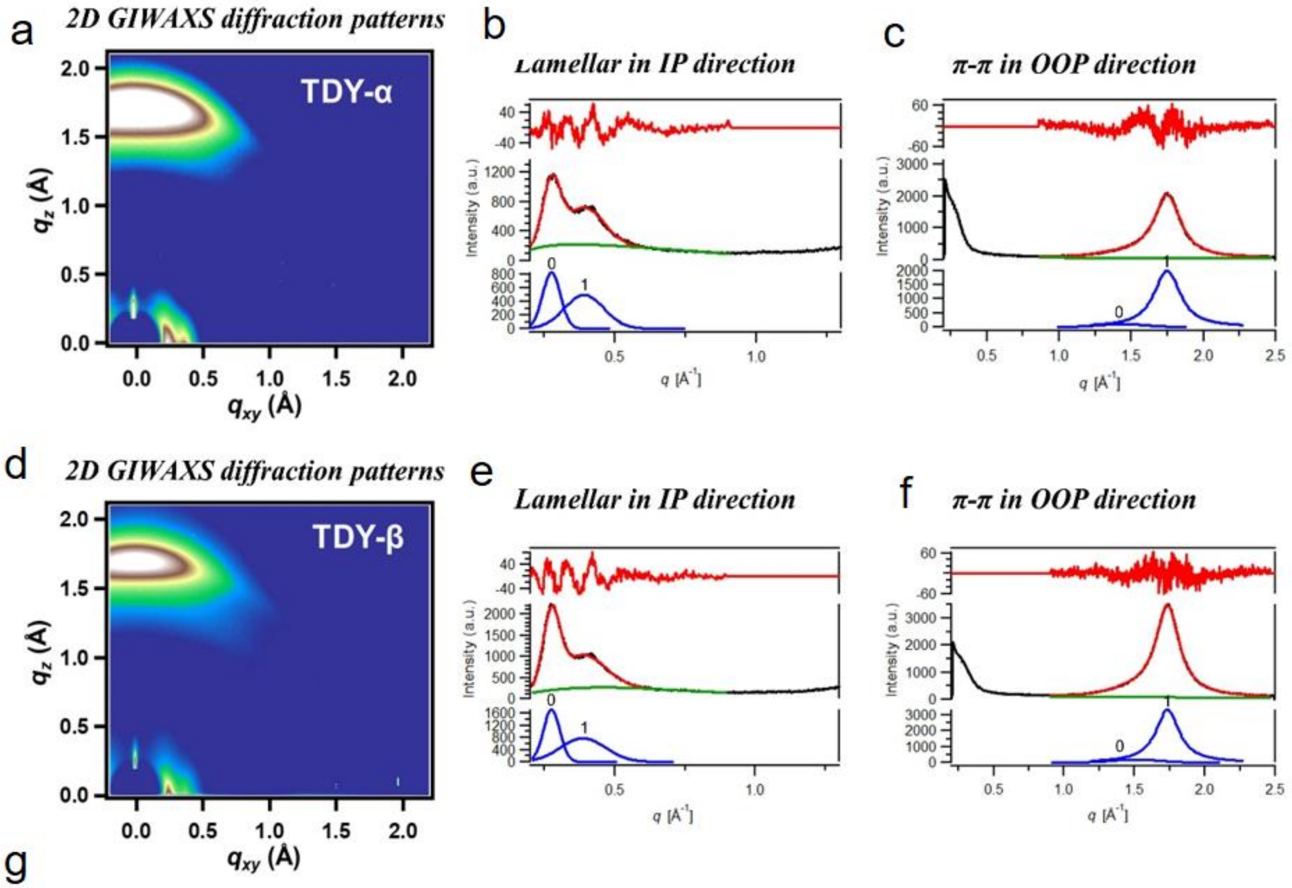


g

| *Lamellar in IP direction* | | $q$ (Å$^{-1}$) | $d$-spacing (Å) | FWHM (Å$^{-1}$) | CCL (Å) |
|---|---|---|---|---|---|
| TDY-α | Peak 0 | 0.28 | **22.44** | 0.079 | **71.58** |
| | Peak 1 | 0.40 | 15.71 | 0.163 | 34.69 |
| TDY-β | Peak 0 | 0.28 | **22.44** | 0.069 | **81.95** |
| | Peak 1 | 0.39 | 16.11 | 0.185 | 30.57 |
| ***π-π in OOP direction*** | | $q$ (Å$^{-1}$) | $d$-spacing (Å) | FWHM (Å$^{-1}$) | CCL (Å) |
| TDY-α | | 1.75 | **3.59** | 0.22 | **25.70** |
| TDY-β | | 1.75 | **3.62** | 0.20 | **28.27** |

**Supplementary Figure 16.** Peak-fitting results of the GIWAXS. The 2D GIWAXS diffraction patterns of (a) TDY-α and the corresponding peak-fitting of 1D profiles in (b) IP and (c) OOP directions. The 2D GIWAXS diffraction patterns of (b) TDY-α and the corresponding peak-fitting of 1D profiles in (e) IP and (f) OOP directions. (g) The summarized results in table.

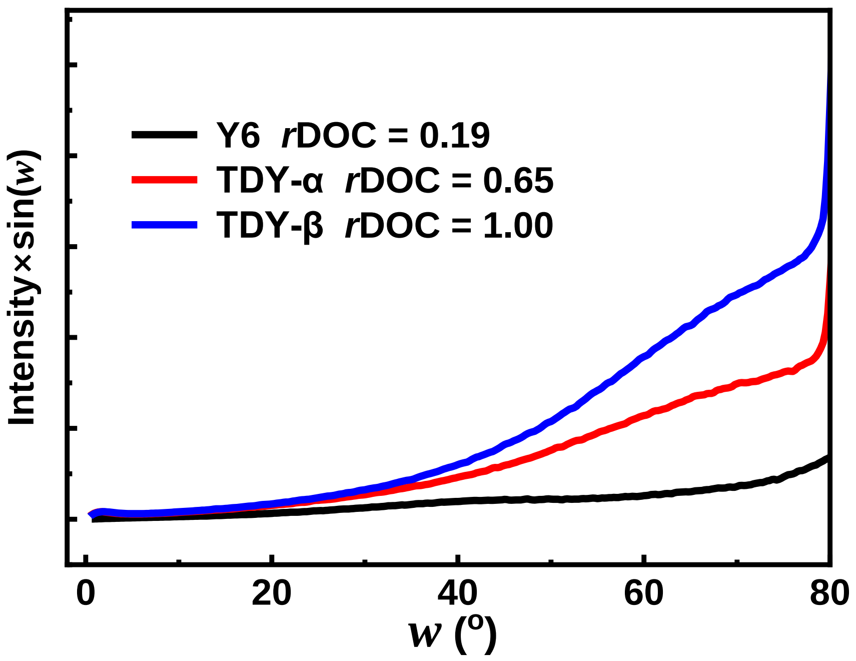


**Supplementary Figure 17.** The calculation of the relative degree of crystallinity (rDOC) of the three acceptors according to the GIWAXS.

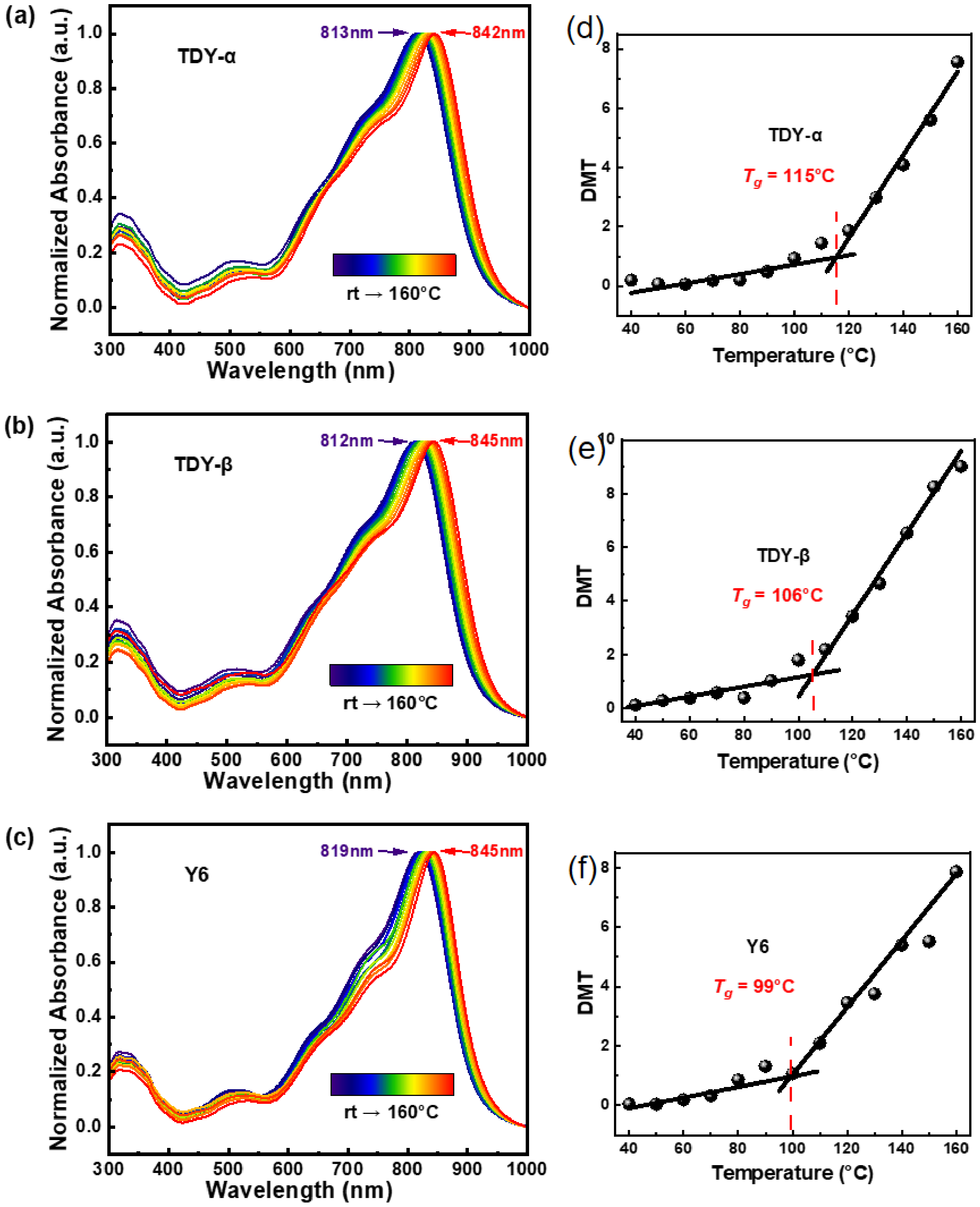


**Supplementary Figure 18.** Derived $T_g$ values. Normalized absorption spectra of (a) TDY-α, (b) TDY-β and (c) Y6 thin films at temperatures varied from room temperature to 160 °C, and the corresponding deviation metric and *T*g values of (d) TDY-α, (e) TDY-β and (f) Y6 thin films.

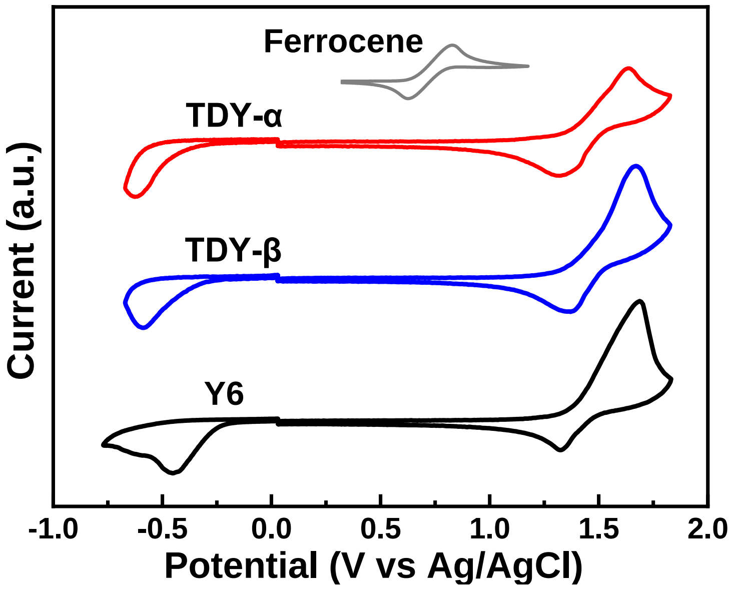


**Supplementary Figure 19.** Cyclic voltammograms of the TSMAs and Y6 films. The $E_{\mathrm{HOMO}}$ / $E_{\mathrm{LUMO}}$ values are estimated to be -5.69 eV/ -3.92 eV, -5.76 eV/-3.96 eV for TDY-α and TDY-β, respectively. With a linker for the dimers, a loser packing of the Y6 subunits in solid to inhibit their initial packing of the Y6 subunits. And as a result, the delocalization of electrons over the π-system may be affected, thus causing an increased bandgap. Moreover, as the dimers favors a folded geometry. According to Kasha's model, the band gap of organic materials is correlated with the slip angle of the two stacked molecules. And the slip angle of the Y6 subunits in the dimers was larger than that of two single Y6 molecules presented in its crystal structure, which may explain the increased bandgap of the dimers.

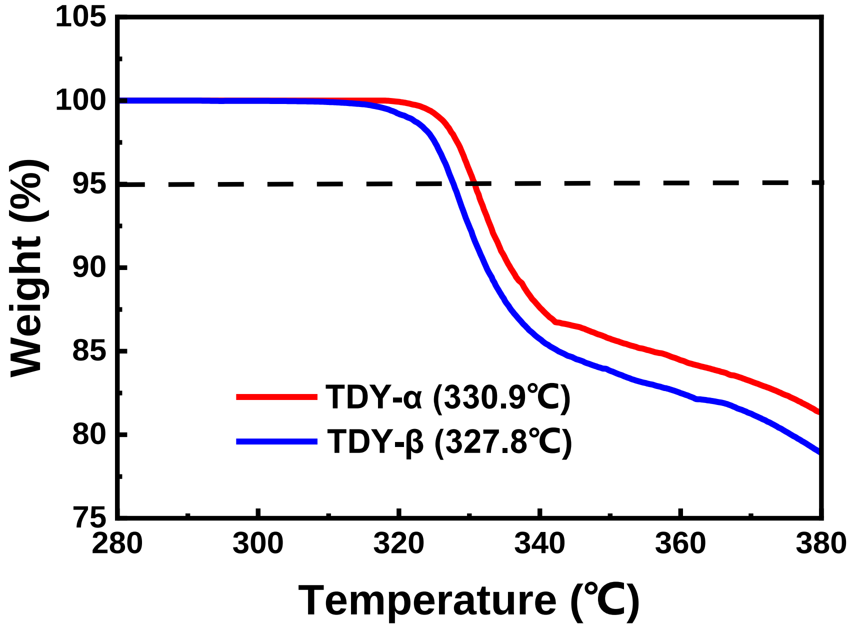


**Supplementary Figure 20.** Thermogravimetric analysis curve of TDY-α and TDY-β with heating rate of 20 K $min^{-1}$.

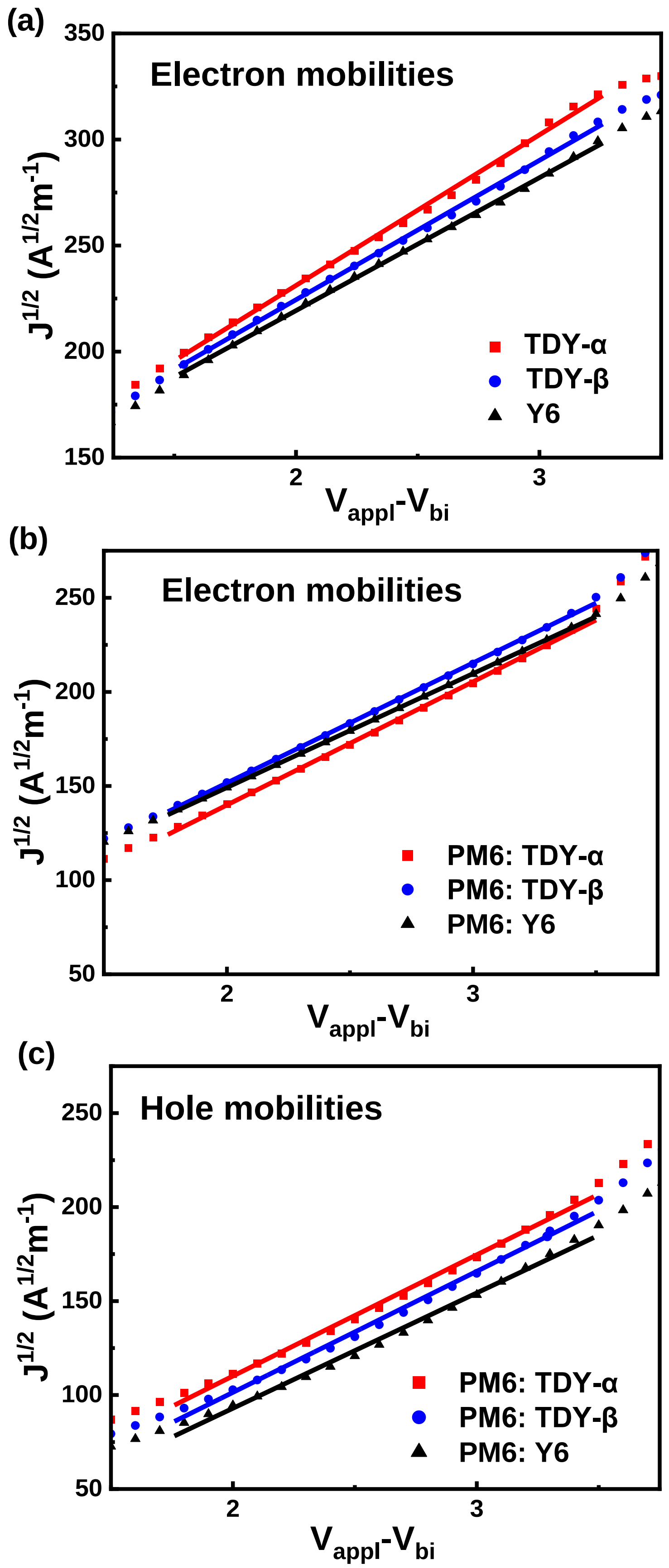


**Supplementary Figure 21.** Mobilities of the dimers and their blends. (a) Electron mobilities of TSMAs. (b) Electron mobilities and (c) hole mobilities of blend films.

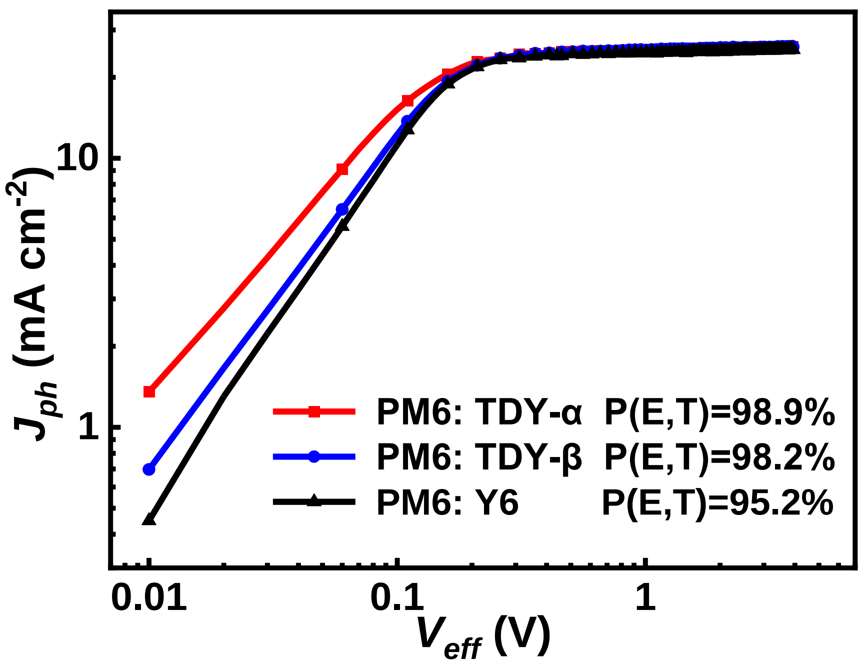


**Supplementary Figure 22.** Light intensity dependence of $V_{oc}$ and $J_{sc}$ of the PSCs. To gain insights on exciton dissociation and charge collection behavior of the PSCs, the charge dissociation probability (P(E,T)) is estimated with relationship between photo current density ($J_{ph}$) and the effective voltage ($V_{eff}$) of the TSMAs-based devices. The photocurrent density $J_{ph}$ is defined as $J_{ph} = J_L - J_D$, where $J_L$ and $J_D$ are the photocurrent densities under AM 1.5G illumination and in the dark condition, respectively. The effective voltage $V_{eff}$ is defined as $V_{eff} = V_0 - V_{bias}$, where $V_0$ is the voltage at which $J_{ph}$ is zero and $V_{bias}$ is the applied external voltage bias. It can be seen that, the increase in $V_{eff}$ results in a higher internal electric field in the device; accordingly, the charge recombination will be minimized. In our cases, $J_{ph}$ tends to an approximate saturation value ($J_{sat}$) at an adequate high $V_{eff}$ of 2.0 V. Thus the charge dissociation probability (P(E,T)) can be estimated from equation of P(E,T) = $J_{ph}$ / $J_{sat}$. Under the short-circuit conditions, the TDY-α and TDY-β based devices have higher P(E,T) values over 98%, which are both higher than that of the Y6-based device (95.2%). These findings demonstrate an improvement of exciton dissociation and charge collection process for the TSMAs, which is in line with a higher $J_{sc}$ value in the TSMAs-based devices.

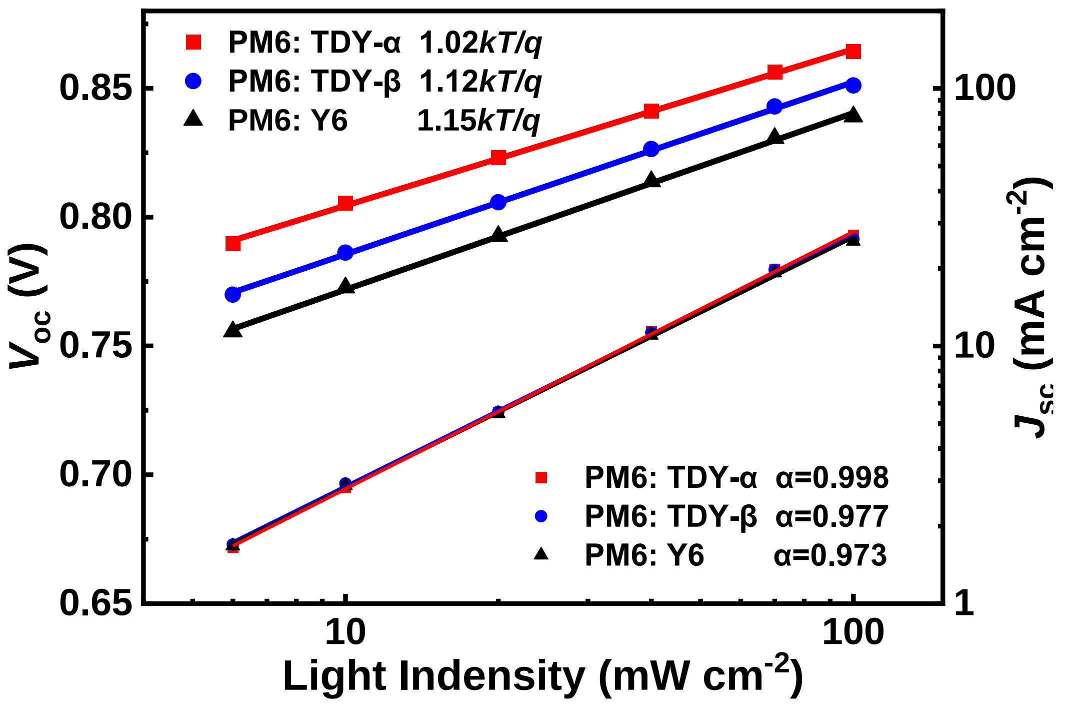


**Supplementary Figure 23.** $J_{ph}$ versus $V_{eff}$ of the optimized devices. The charge recombination behavior of the PSCs is investigated by the dependence of $V_{oc}$ and $J_{sc}$ on light intensity ($P_{light}$). The degree of trap-assisted recombination can be estimated by the relationship of $V_{oc} \propto nk\mathrm{T}/q\ln(P_{light})$. Here, $n$ represents the ideality factor, $k$ is the Boltzmann constant, T is absolute temperature, and $q$ is the elementary charge. If the slope is $k\mathrm{T}/q$, it suggests that bimolecular recombination is the main recombination. And if the slope is close to 2 $k\mathrm{T}/q$, it indicates serious trap-assisted recombination46. As shown in Figure S16, the slopes of the TDY-α, TDY-β and Y6-based devices are 1.02 $k\mathrm{T}/q$, 1.12 $k\mathrm{T}/q$, and 1.15 $k\mathrm{T}/q$, respectively. The smallest slope indicates the TSMA-based devices possess less trap-assisted recombination. On the other hand, the relationship between $J_{sc}$ and $P_{light}$ can be expressed as $J_{sc} \propto (P_{light})^{\alpha}$, which reveals charge bimolecular recombination in devices. The fitting α values of the PSCs based on PM6: TDY-α, PM6: TDY-β, and PM6: Y6 are 0.998, 0.977 and 0.973, respectively. The highest α (closer to 1) demonstrates that there is effective carrier collection and negligible bimolecular recombination in the best-performing TDY-α based devices at the short-circuit condition, which can also explain the best $J_{sc}$ and FF of the TDY-α based devices.

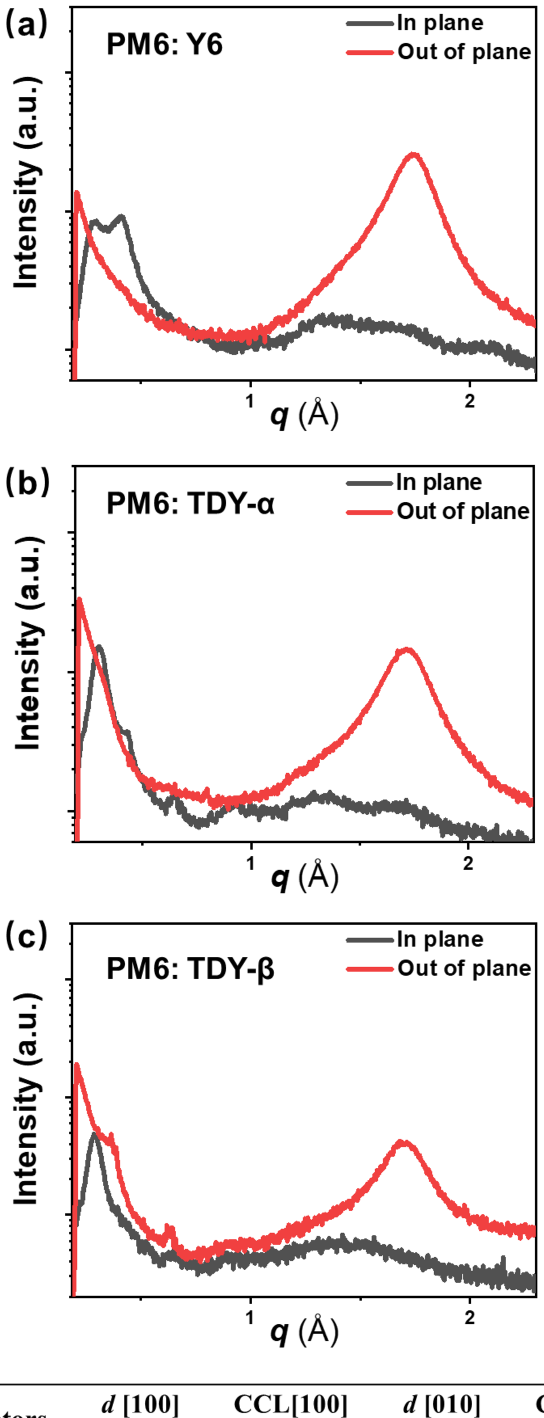


(d)

| Acceptors | *d* [100] (Å) | CCL[100] (Å) [a] | *d* [010] (Å) | CCL[010] (Å) [a] |
|---|---|---|---|---|
| TDY-α | 20.87 | 102.81 | 3.68 | 25.70 |
| TDY-β | 21.44 | 97.49 | 3.71 | 24.91 |
| Y6 | 21.82 | 73.44 | 3.60 | 24.80 |

**Supplementary Figure 24.** 2D-GIWAXS patterns of the blend films. Line-cuts of 2D-GIWAXS patterns in the in-plane and out-of-plane directions of (a) PM6: TDY-α, (b) PM6: TDY-β and (c) PM6: Y6 blends films after thermal annealing for 5 min. (d) The crystallinity properties of the PM6: Acceptors blend films after annealing at 100 ℃ for 5 min.

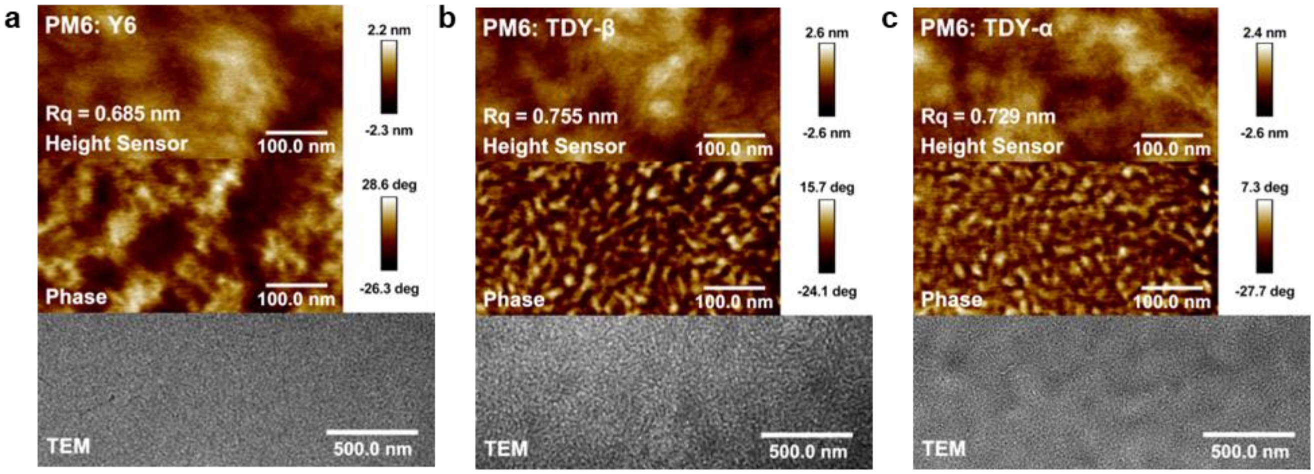


**Supplementary Figure 25.** Morphology of the blend films. AFM height images, phase images and corresponding TEM images of the blend films for (a) PM6: TDY-α, (b) PM6: TDY-β, and (c) PM6: Y6.

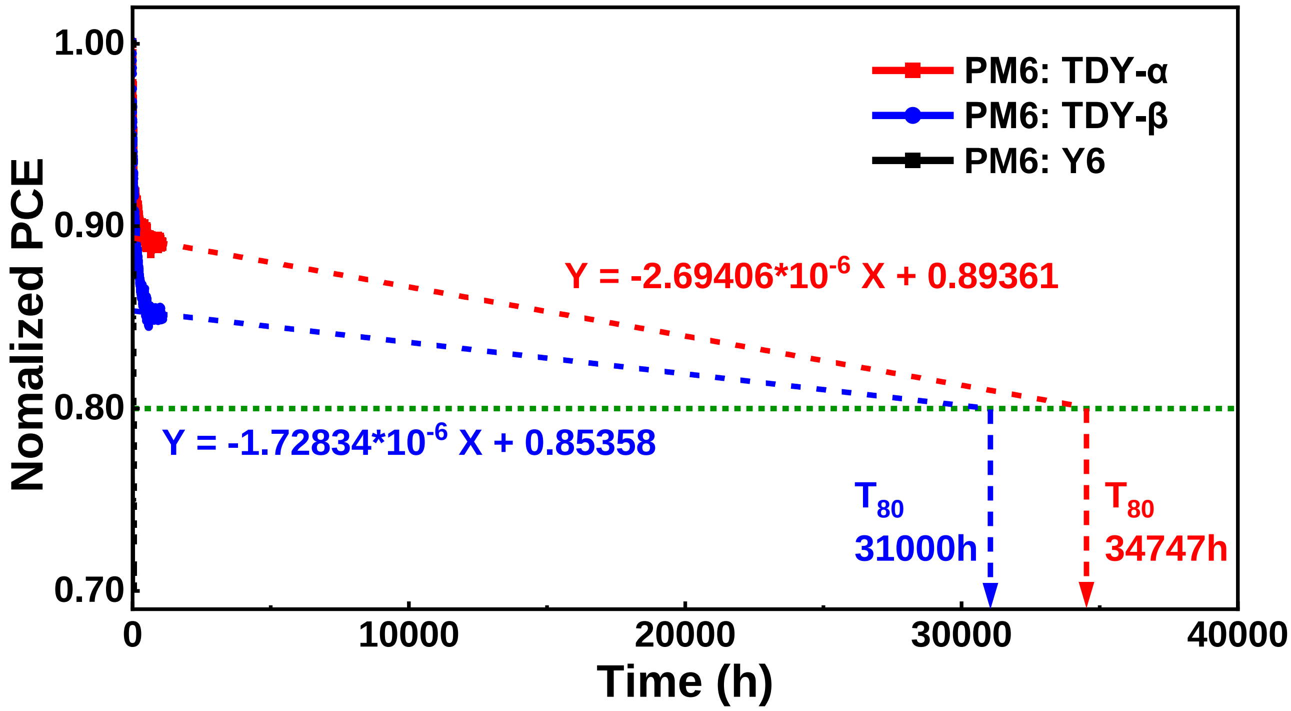


**Supplementary Figure 26.** MPP stability test of the PM6:Y6 and PM6: TSMAs based devices under 1-Sun equivalent illumination from white LEDs at the MPP conditions in open-air. The dashed line corresponds to the linear fitting result from 600 h to 1100 h, excluding the influence of burn-in loss.

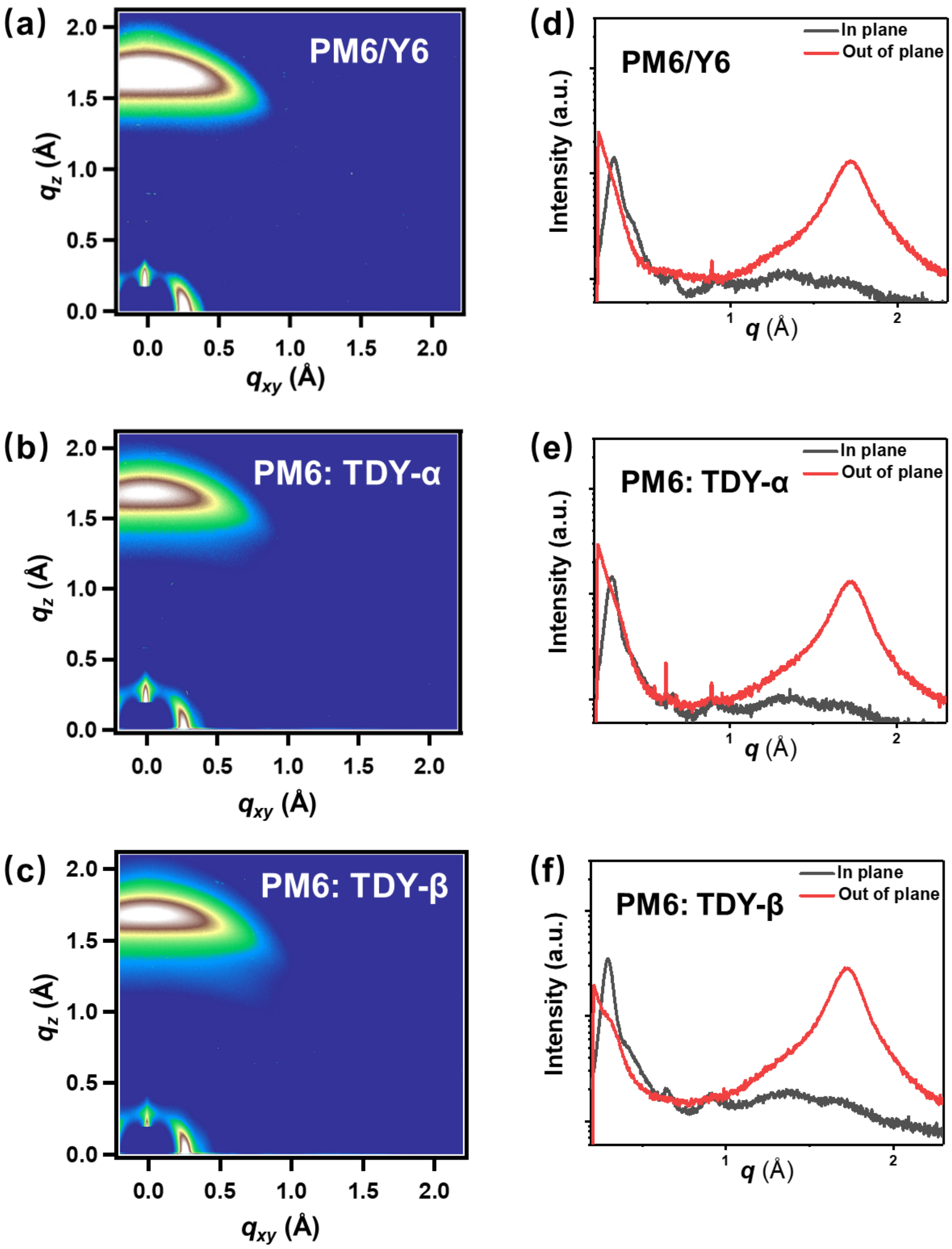


(g)

| Acceptors | *d* [100] (Å) | CCL[100] (Å) | *d* [010] (Å) | CCL[010] (Å) |
|---|---|---|---|---|
| TDY-α | 21.37 | 104.72 | 3.66 | 28.70 |
| TDY-β | 21.37 | 100.98 | 3.66 | 25.36 |
| Y6 | 21.01 | 88.35 | 3.67 | 23.56 |

**Supplementary Figure 27.** The 2D GIWAXS diffraction patterns of (a) PM6: TDY-α, (b) PM6: TDY-β and (c) PM6: Y6 blends films after thermal annealing for 4h. Corresponding line-cuts of 2D-GIWAXS patterns in the in-plane and out-of-plane directions of (d) PM6: TDY-α, (e) PM6: **TDY-β** and (f) PM6: Y6 blends films. (g) The crystallinity properties of PM6/acceptors blend films after annealing at 100 °C for 4 h.

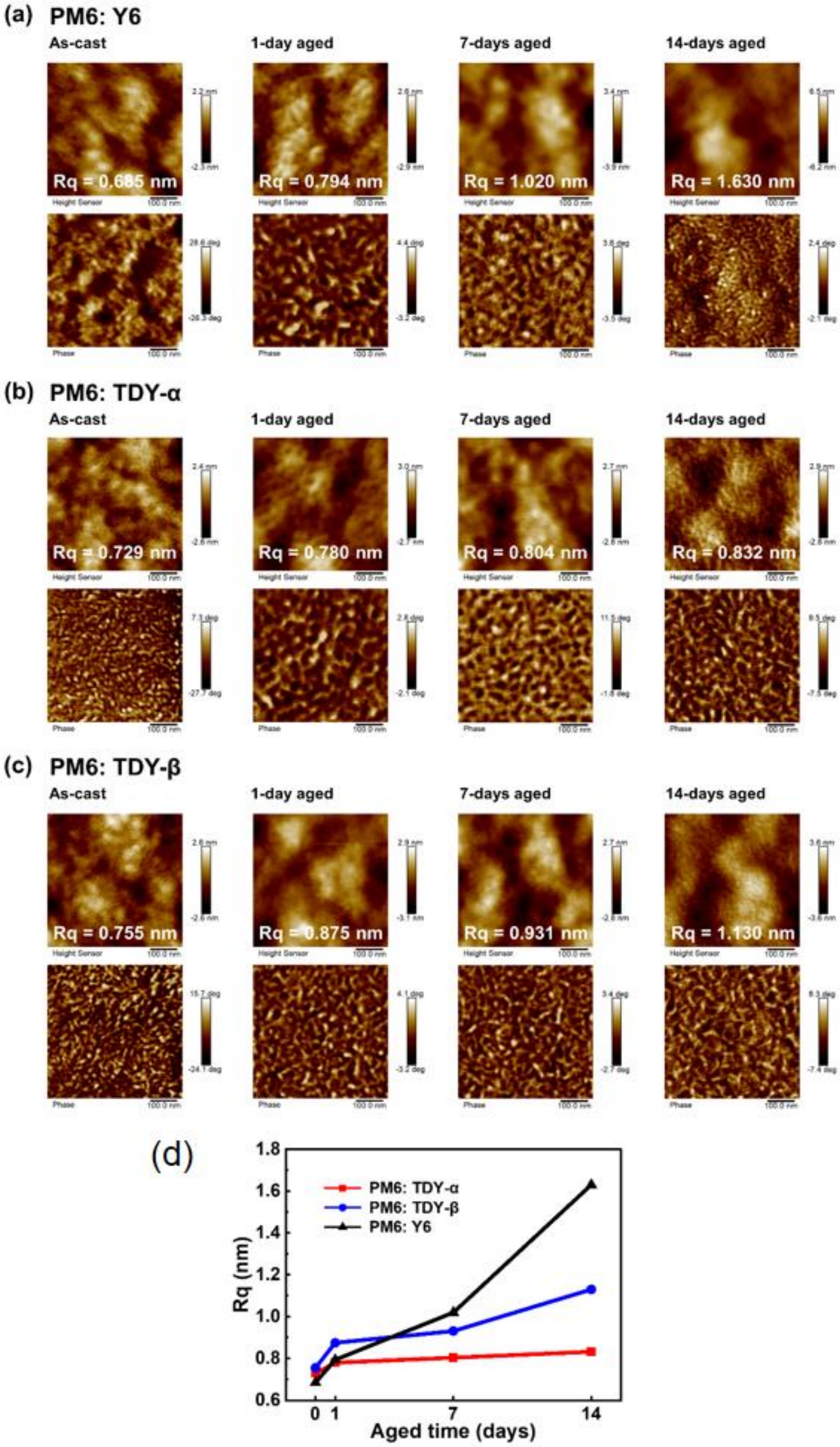


**Supplementary Figure 28.** Morphology of the blend films. AFM height and phase images of (a) PM6: Y6, (b) PM6: TDY-α, (c) PM6: TDY-β of the as-cast blend films and thermal aged blend films annealed at 85 ℃ for 1, 7 and 14 days. (d) Corresponding Rq values diagrams of the PM6: Y6 and PM6: TSMAs as-cast blend films and thermal aged blend films annealed at 85 ℃ for 1, 7 and 14 days.

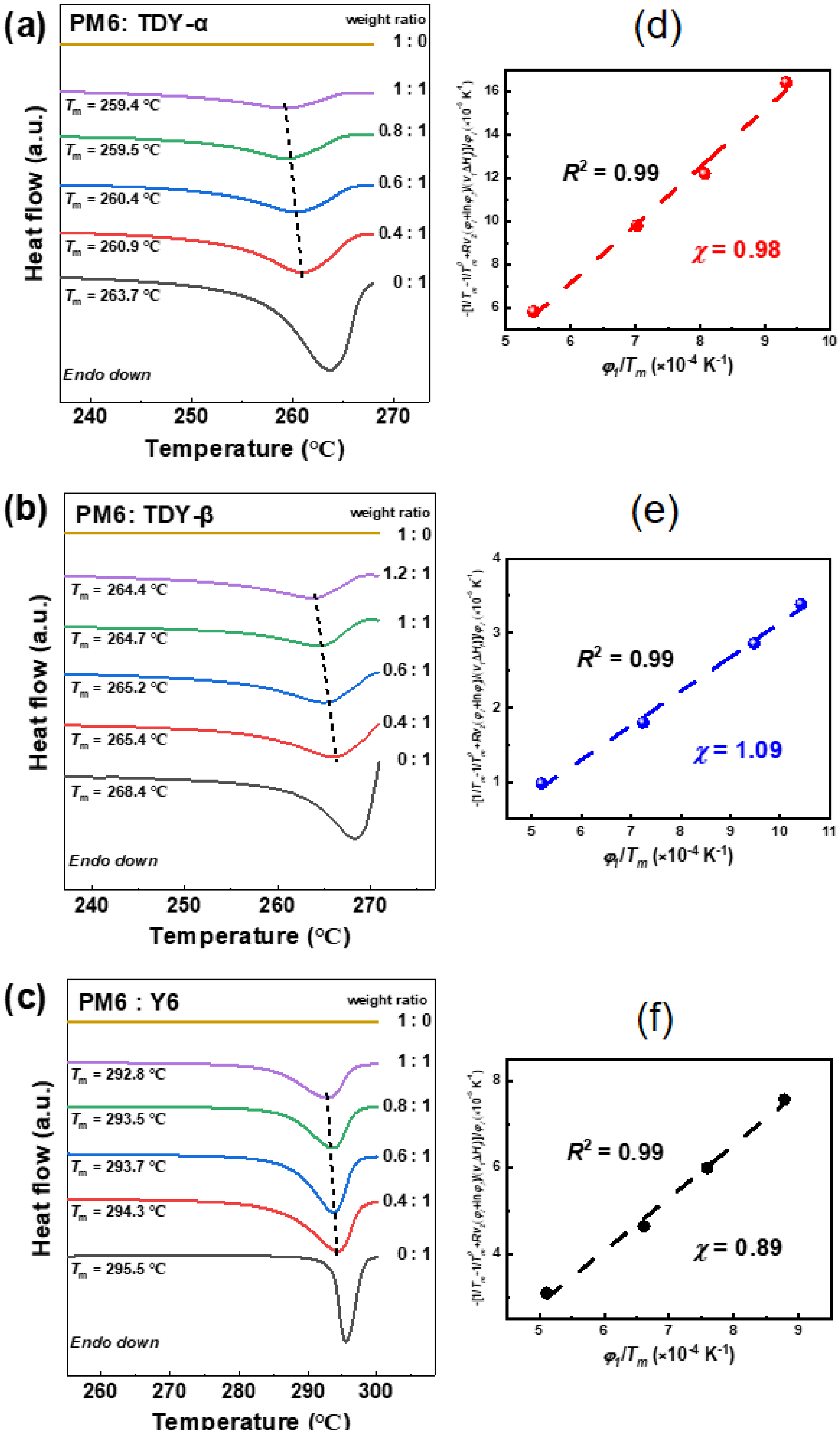


**Supplementary Figure 29.** The calculation of the χ values. DSC thermograms of (a) PM6: TDY-α, (b) PM6: TDY-β, (c) PM6: Y6 under various mass ratios. The estimation of the χ values by the melting point depression from DSC thermograms for (d) PM6: TDY-α, (e) PM6: TDY-β, (f) PM6: Y6 blends.

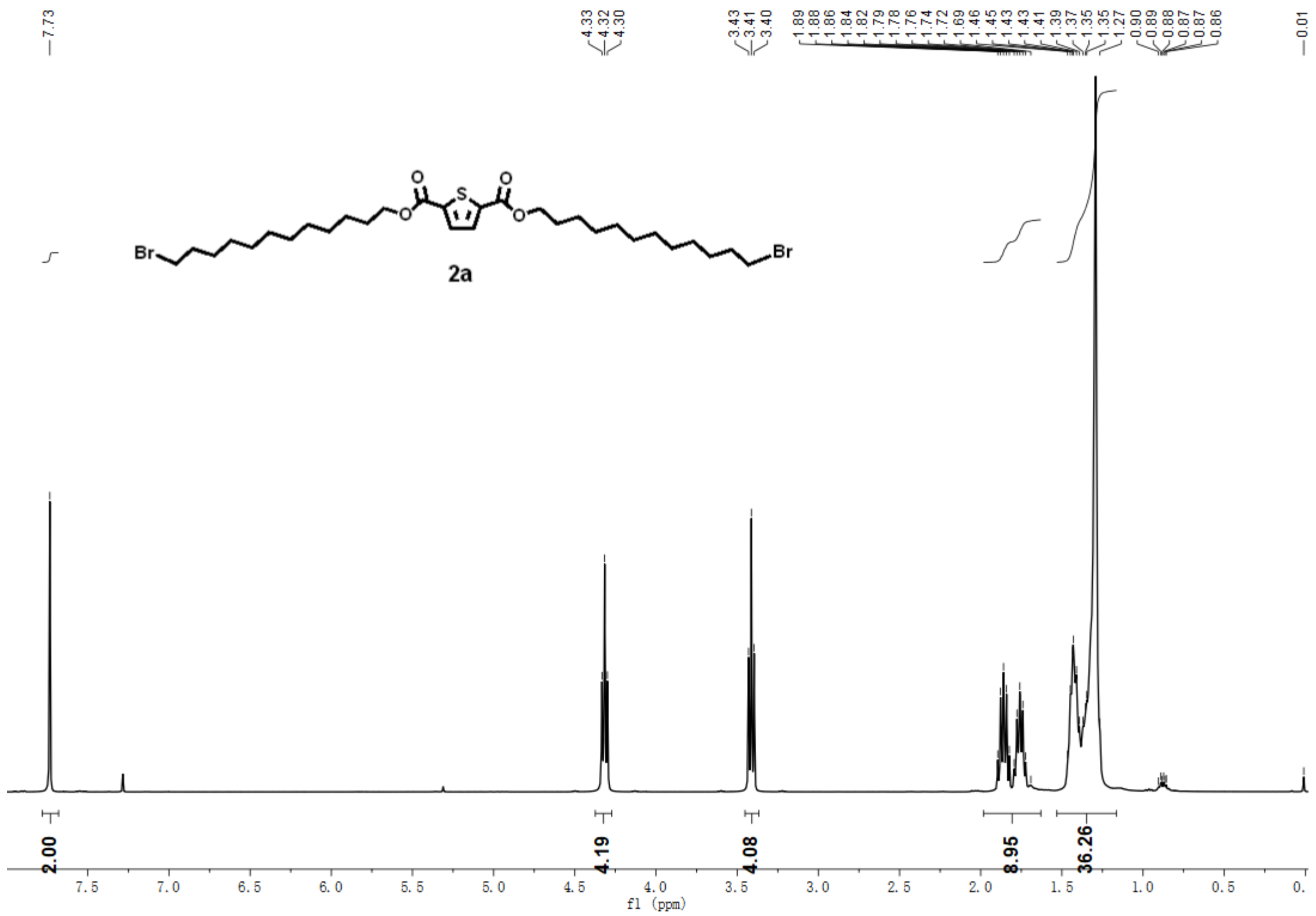


**Supplementary Figure 30.** $^{1}$H NMR spectrum of 2a.

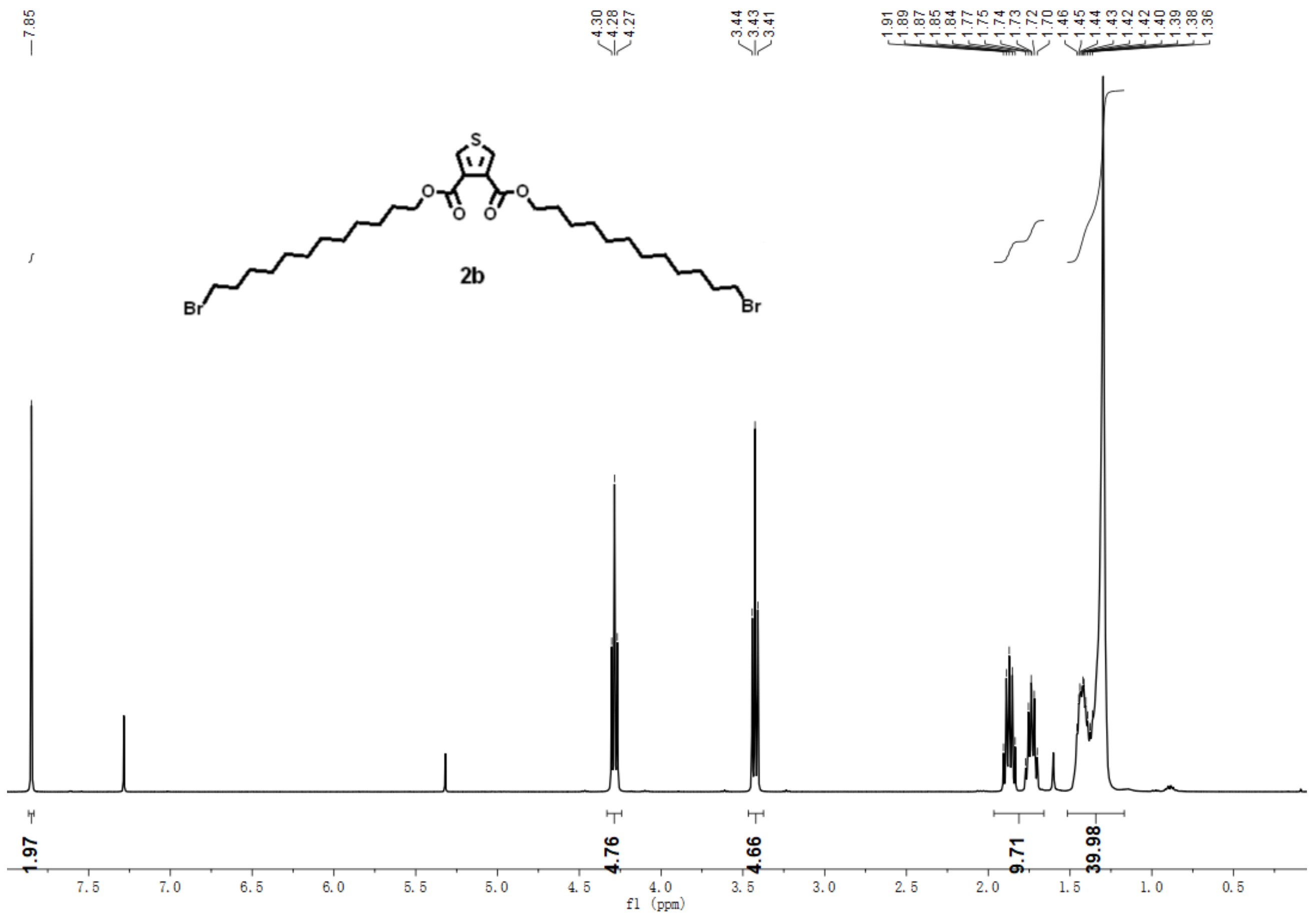

**Supplementary Figure 31.** $^1$H NMR spectrum of 2b.

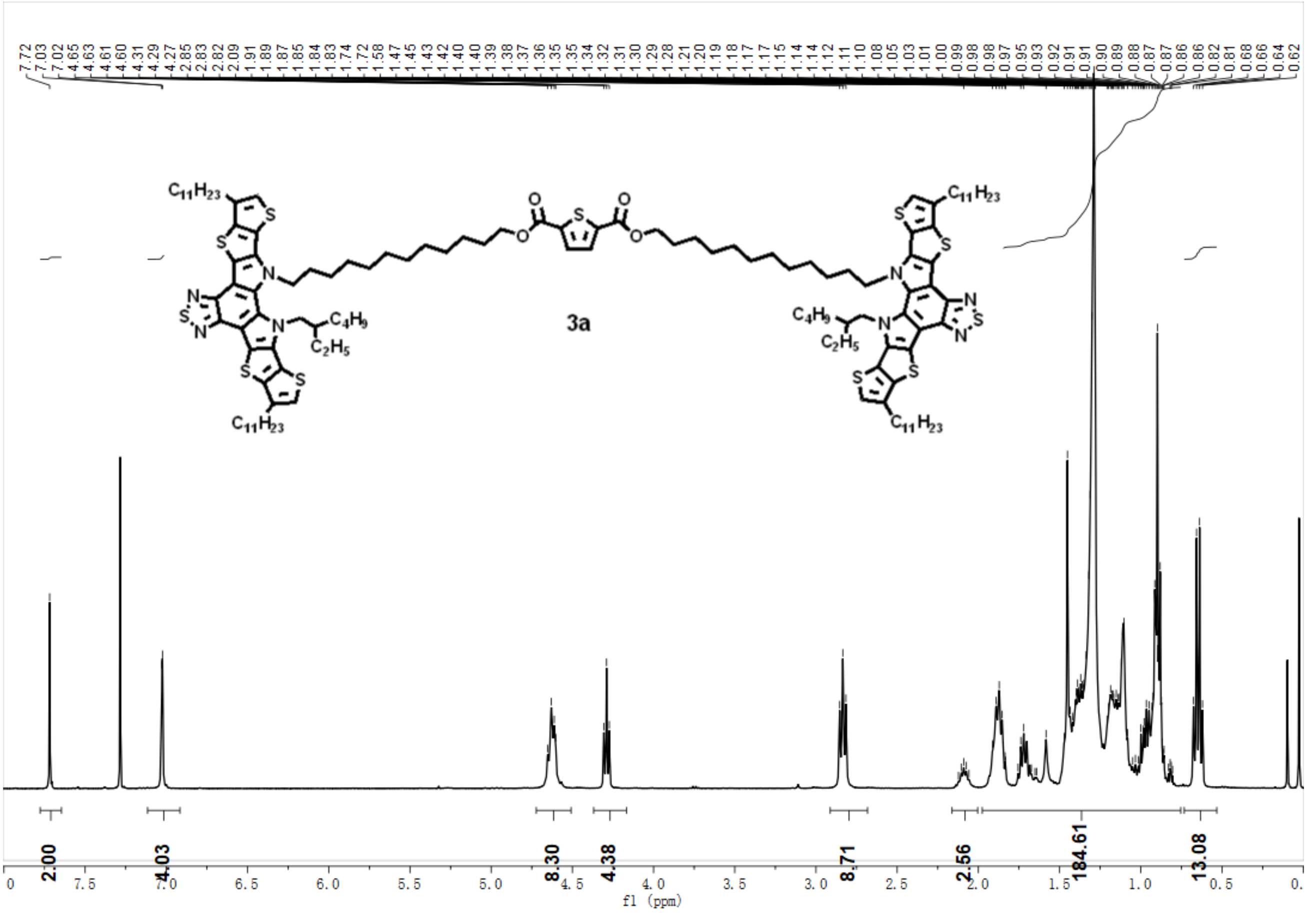

**Supplementary Figure 32.** $^1$H NMR spectrum of 3a.

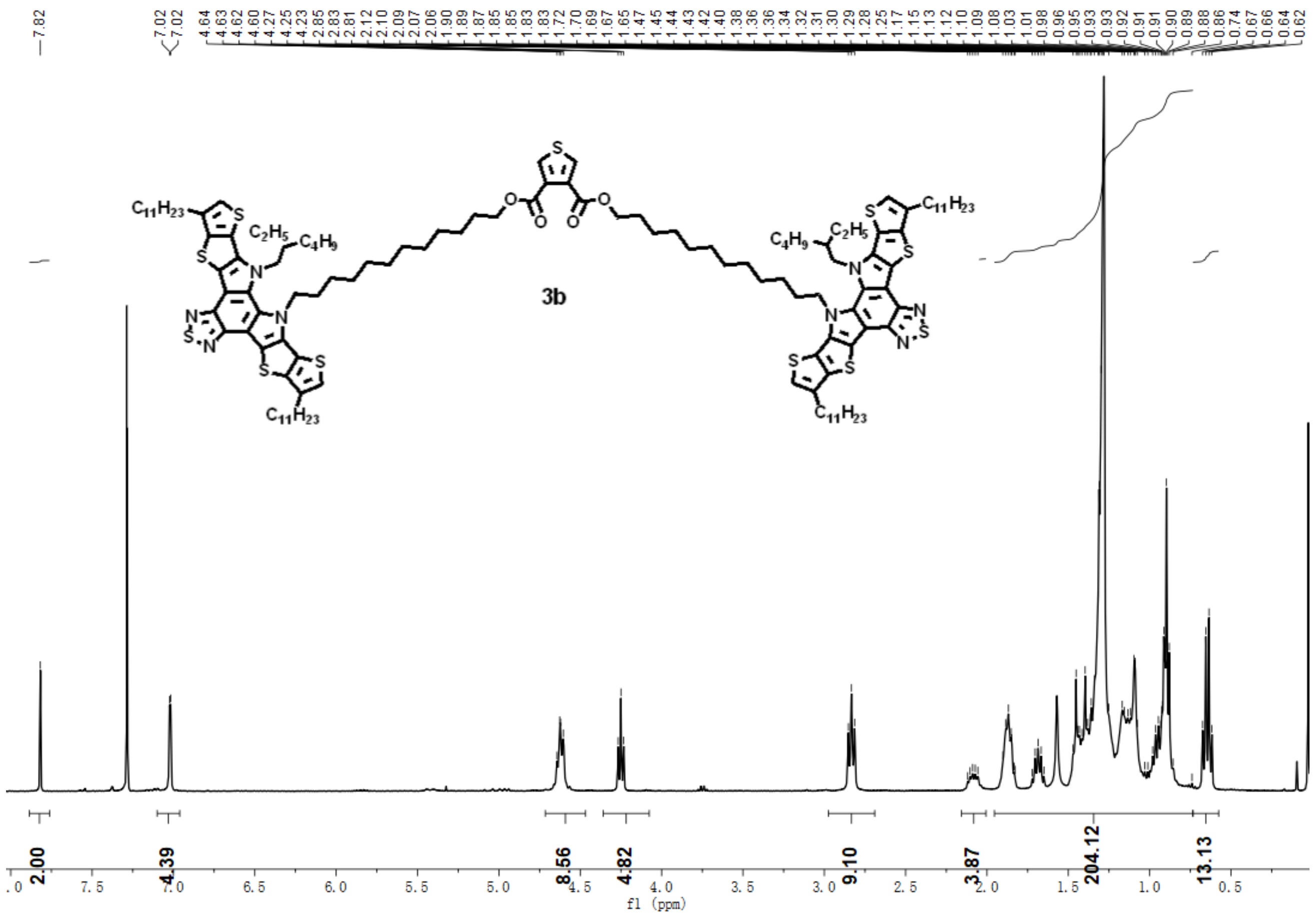

**Supplementary Figure 33.** $^1$H NMR spectrum of 3b.

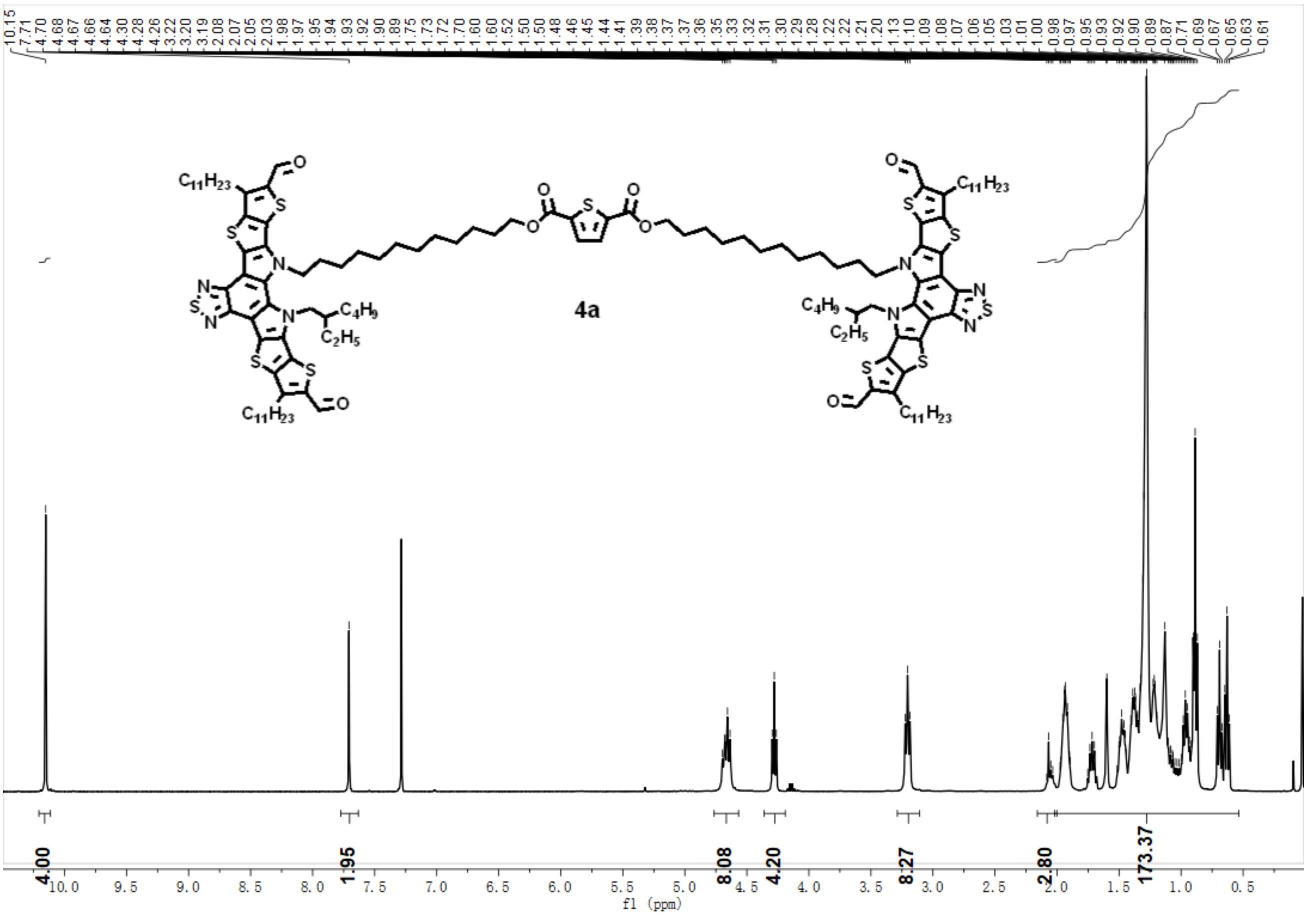

**Supplementary Figure 34.** $^{1}$H NMR spectrum of 4a.

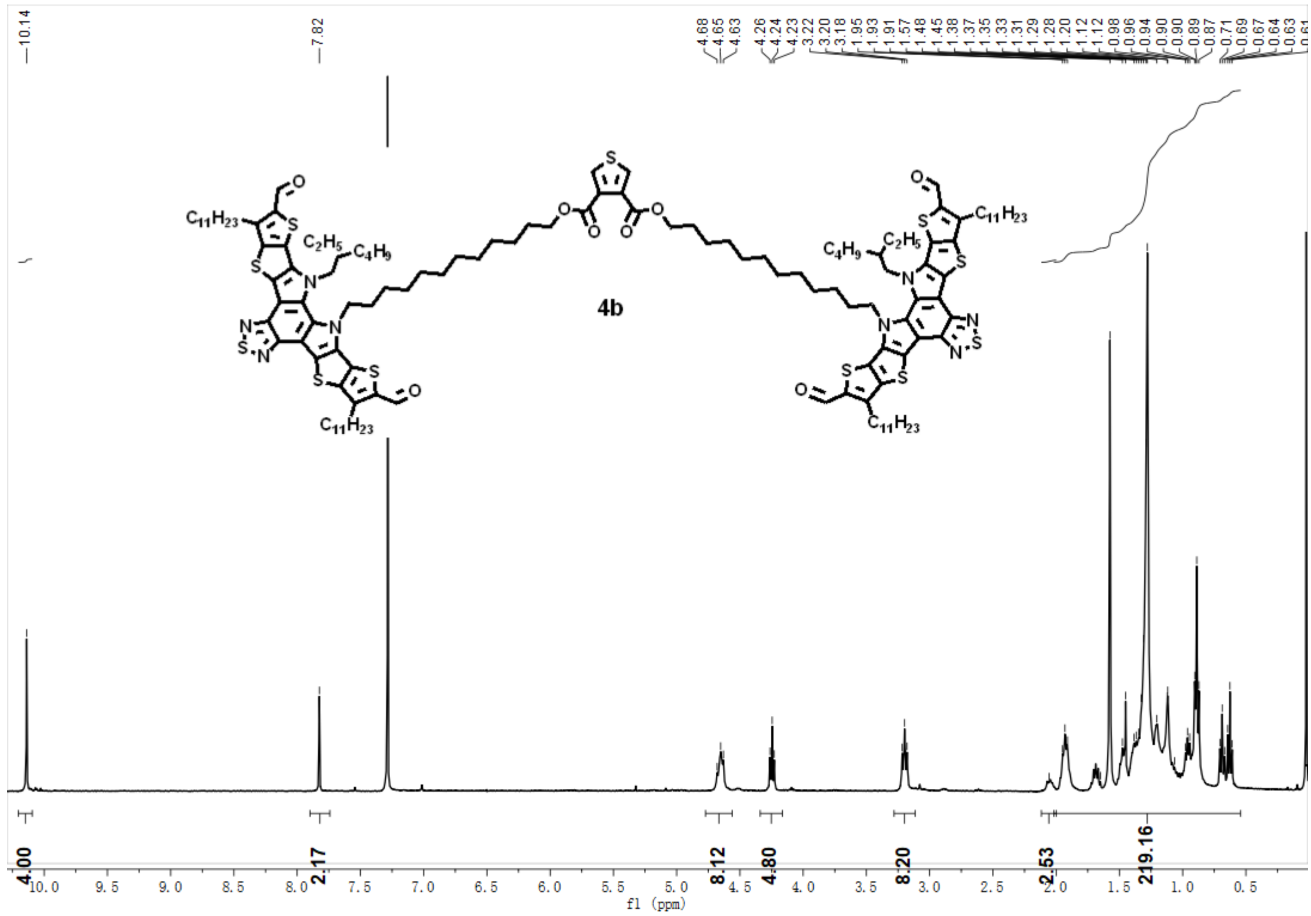

**Supplementary Figure 35.** $^{1}$H NMR spectrum of 4b.

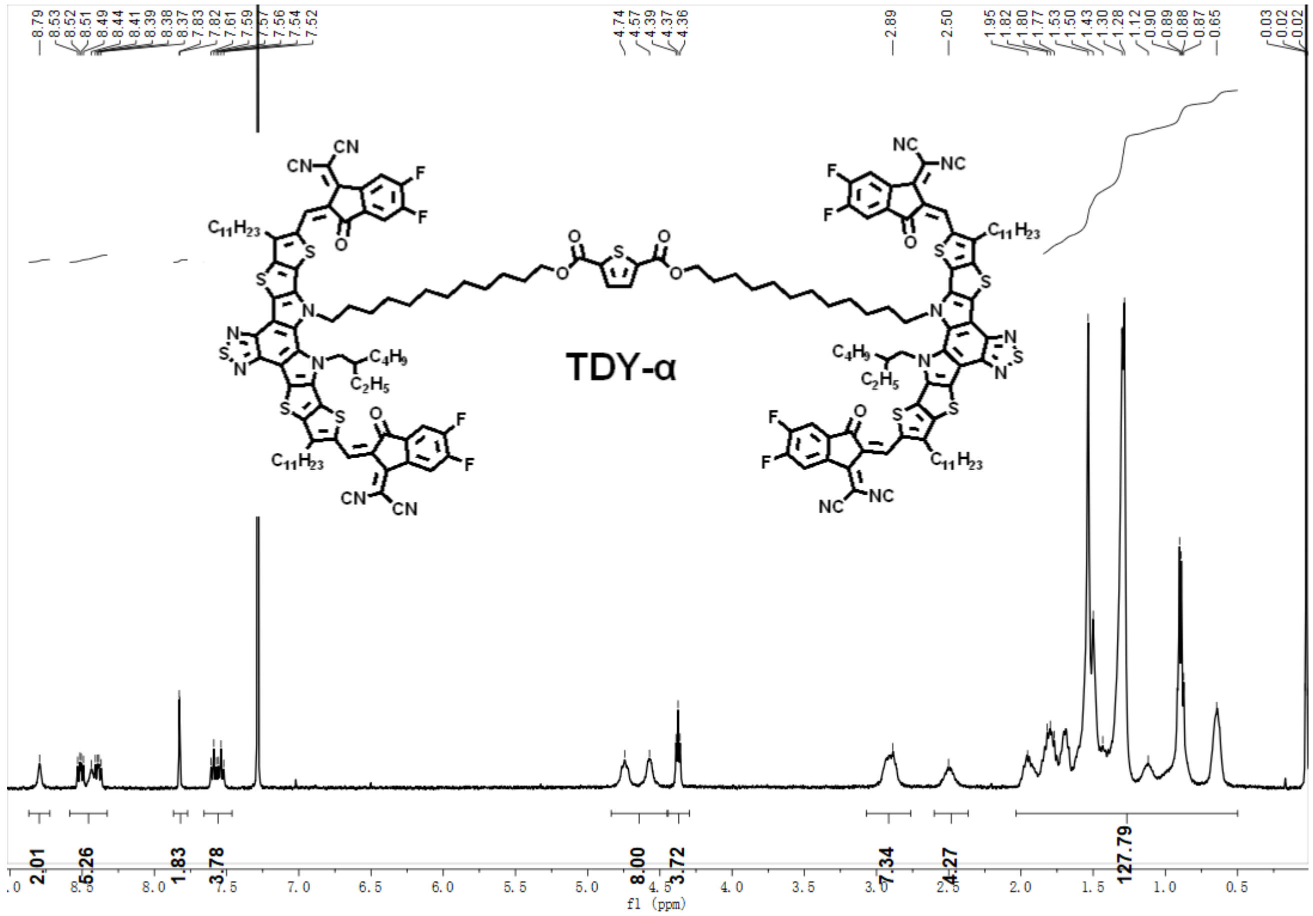

**Supplementary Figure 36.** $^{1}H$ NMR (298K) spectrum of TDY-α.

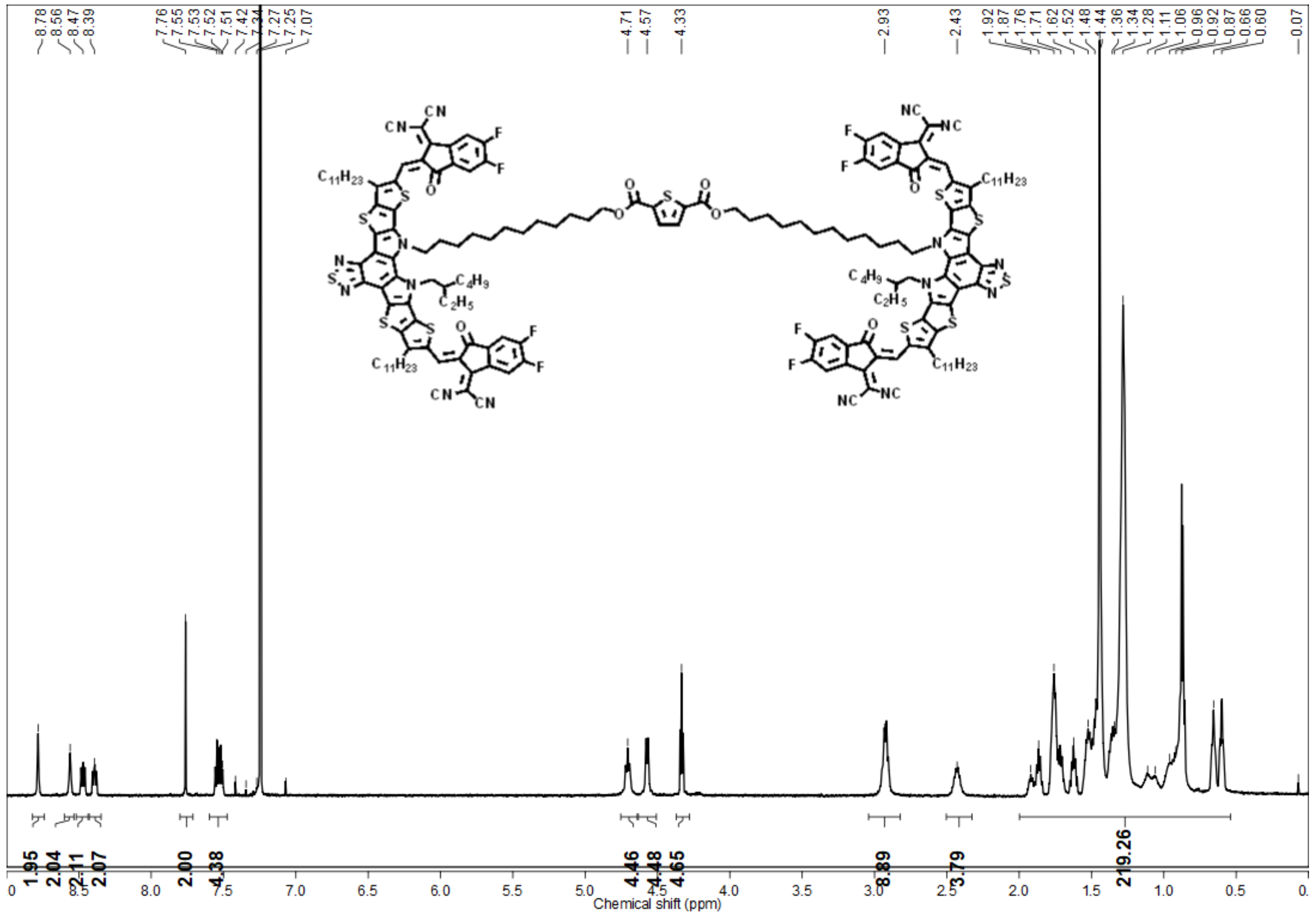

**Supplementary Figure 37.** $^{1}H$ NMR (331K) spectrum of TDY-α.

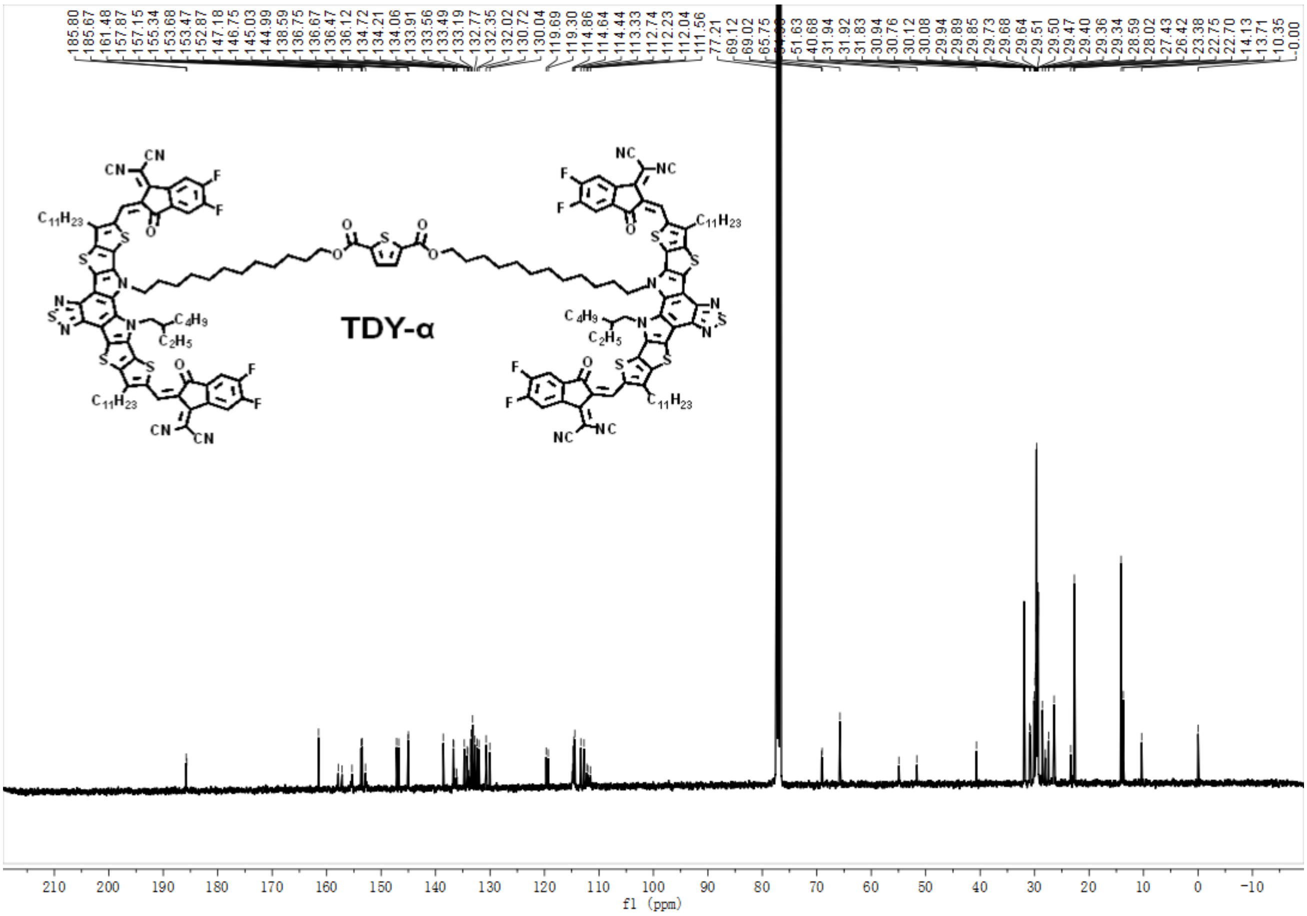

**Supplementary Figure 38.** $^{13}$C NMR spectrum of TDY-α.

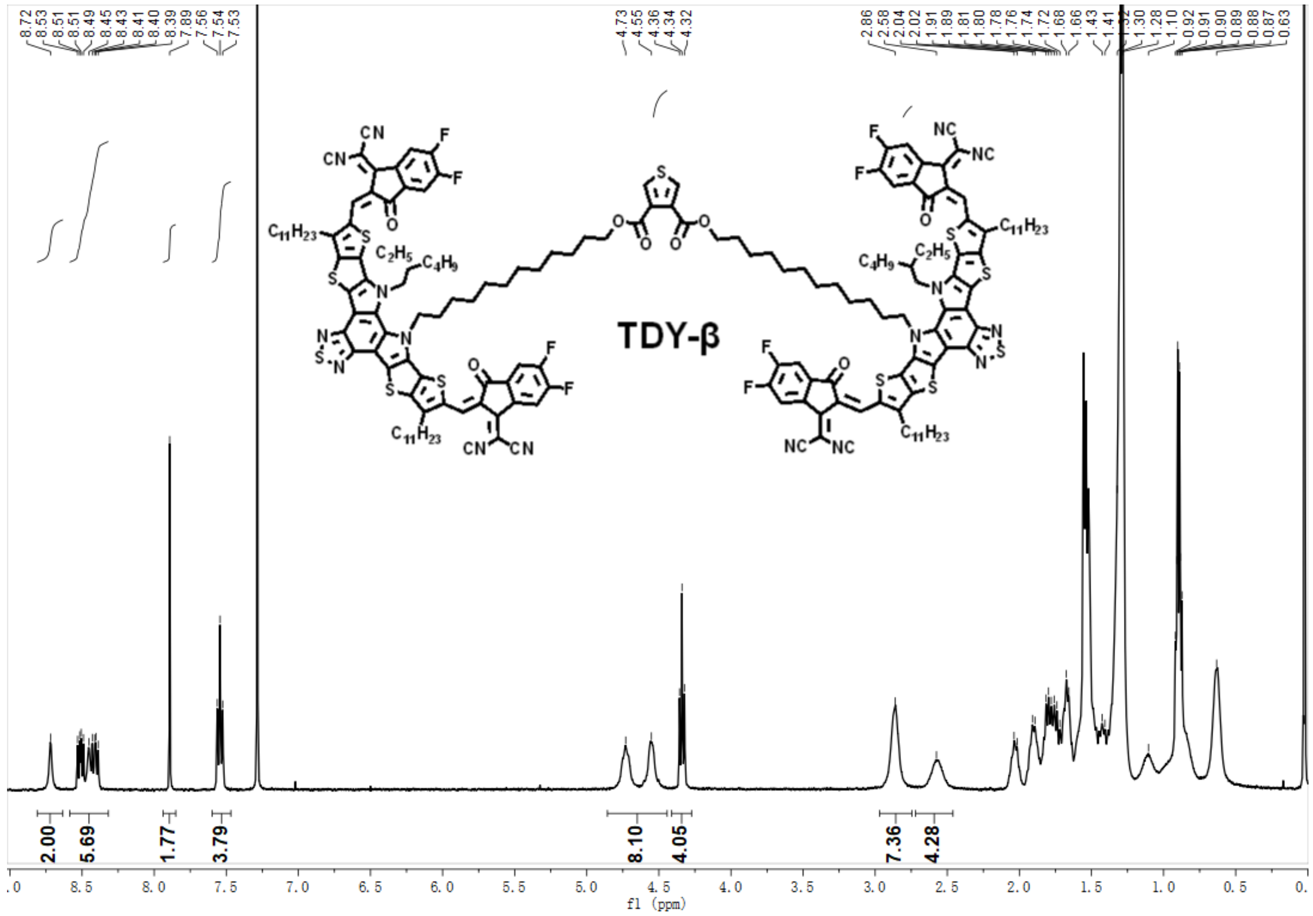

**Supplementary Figure 39.** $^1$H NMR (298K) spectrum of TDY-β.

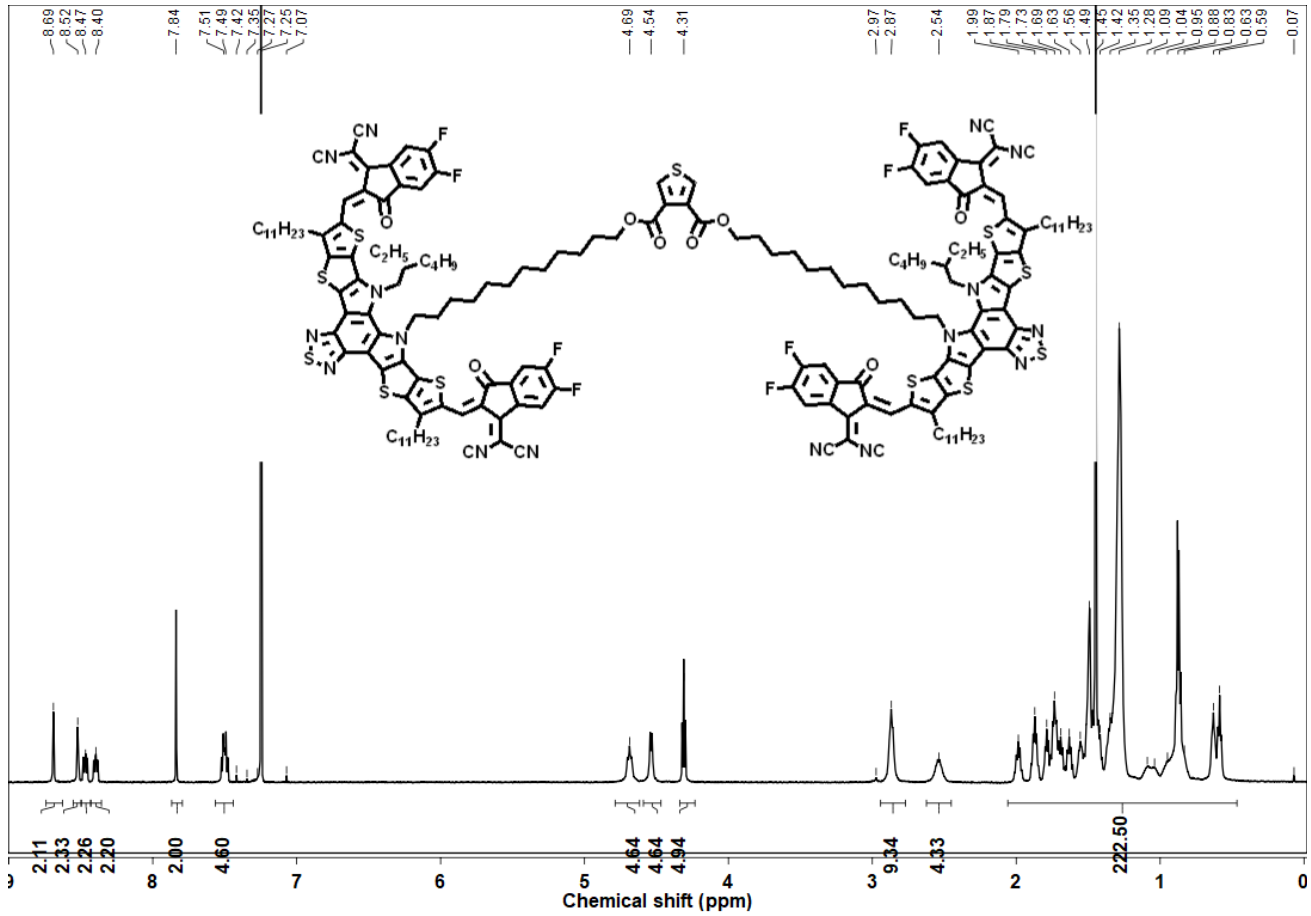

**Supplementary Figure 40.** $^{1}H$ NMR (331K) spectrum of TDY-β.

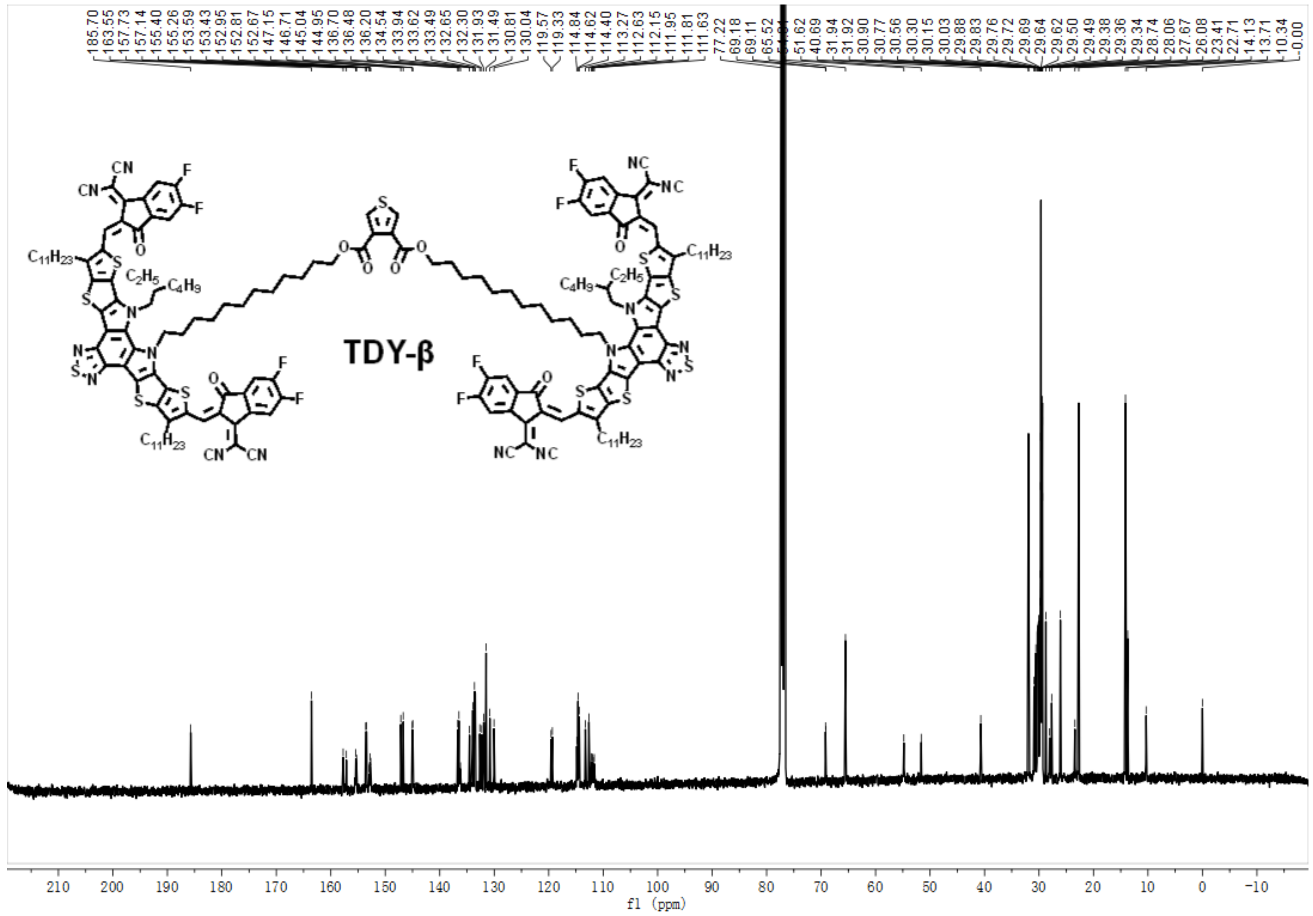

**Supplementary Figure 41.** $^{13}$C NMR spectrum of TDY-β.

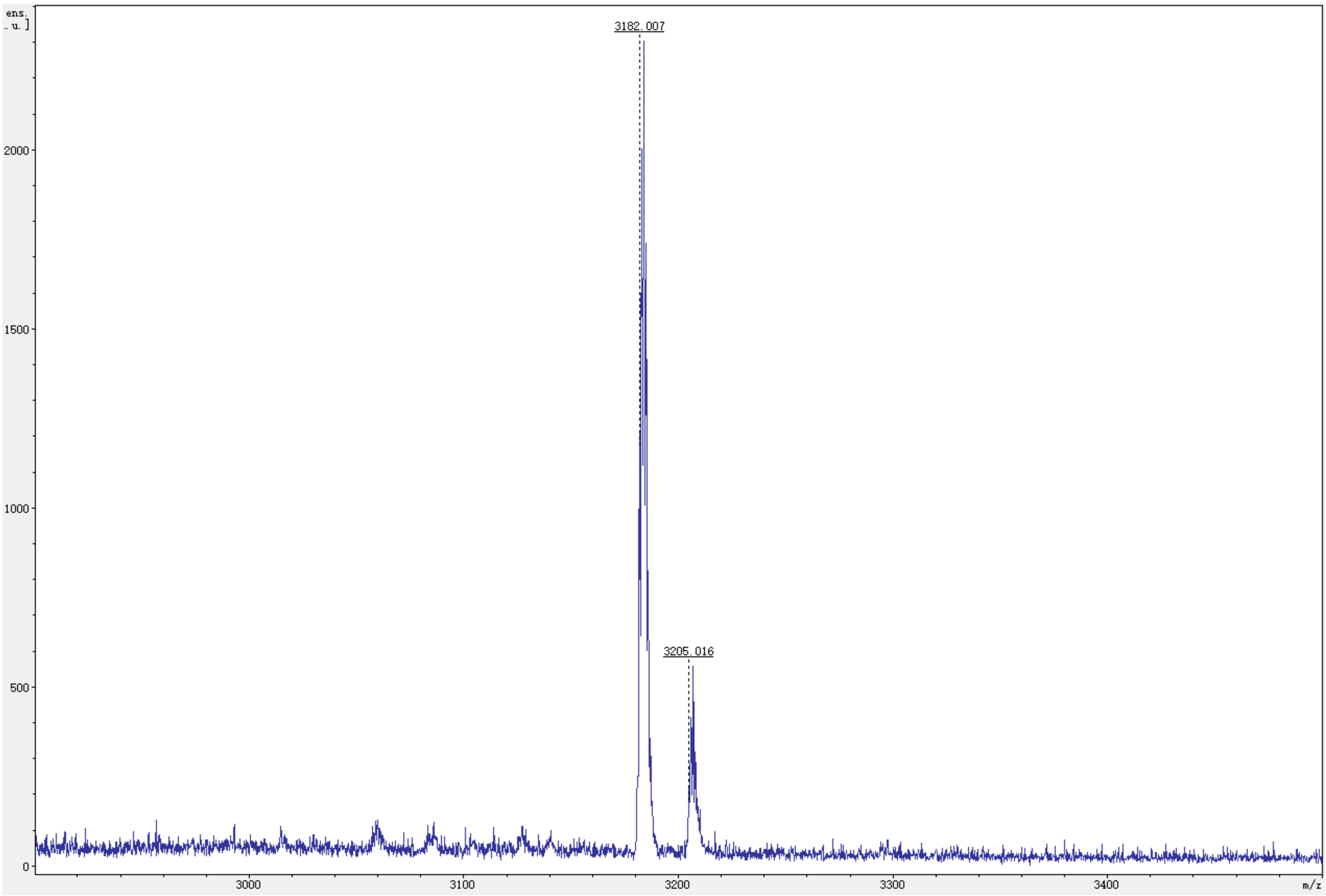


**Supplementary Figure 42.** MS (MALDI-TOF) spectrum of TDY-α.

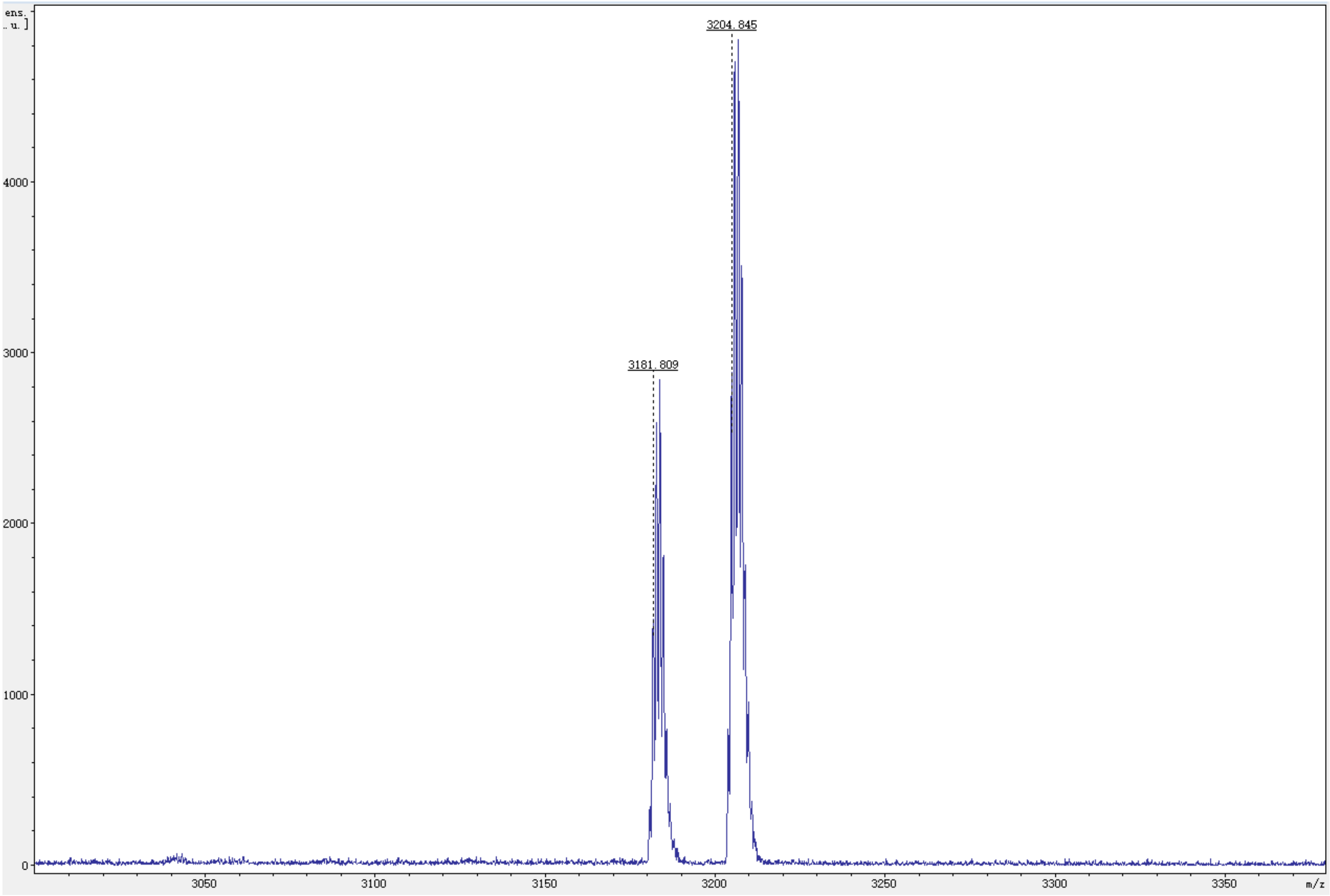


**Supplementary Figure 43.** MS (MALDI-TOF) spectrum of TDY-β.

**Supplementary Table 1**. Photovoltaic parameters of the devices based on PM6: acceptors with D/A weight ratio of 1: 1.2 and thermal annealing at 100 ºC for 5 min. under the illumination of AM1.5G, 100 mW/cm$^2$.[a]

| **Active layer** | **Additive** | **Speed** | **Annealing** |
|---|---|---|---|
| PM6: Y6 | 0.5% CN | 3600 rpm | 90℃ 5min |
| PM6: TDY-α | 1% CN | 3900 rpm | 100℃ 5min |
| PM6: TDY-β | 1% CN | 3900 rpm | 100℃ 5min |

[a] Average values based on ten devices.
