# Supplementary material for: Geometry design of tethered small-molecule acceptor enables highly stable and efficient polymer solar cells
Source: Nat Commun. 2023 May 22;14:2926. doi: 10.1038/s41467-023-38673-5 (PMC10203300; doi:10.1038/s41467-023-38673-5)
Supplement: Supplementary file 3 — Reporting Summary [file 41467_2023_38673_MOESM3_ESM.pdf]

Corresponding author(s): Zhi-Guo Zhang

Solar Cells Reporting Summary

Nature Research wishes to improve the reproducibility of the work that we publish. This form is intended for publication with all accepted papers reporting the characterization of photovoltaic devices and provides structure for consistency and transparency in reporting. Some list items might not apply to an individual manuscript, but all fields must be completed for clarity.

For further information on Nature Research policies, including our data availability policy, see Authors & Referees.

ü Experimental design

Please check: are the following details reported in the manuscript?

1. Dimensions

Item	Reported	Details
Area of the tested solar cells	[x] Yes [ ] No	Area of the tested solar cells is 0.06 cm2, it is provided in the main text, section of "Device Fabrication"
Method used to determine the device area	[ ] Yes [x] No	Active area of 0.06 cm2 was comfirmed by aperture and devices were tested without mask

2. Current-voltage characterization

Item	Reported	Details
Current density-voltage (J-V) plots in both forward and backward direction	[ ] Yes [x] No	Only the plots in forward direction was supplied.
Voltage scan conditions<br>*For instance: scan direction, speed, dwell times*	[ ] Yes [x] No	The voltage step and delay time were 10 mV and 1 ms, respectively. The scan started from −0.2 V to 1.0 V
Test environment<br>*For instance: characterization temperature, in air or in glove box*	[ ] Yes [x] No	The solar cells were tset in glove box at 25℃.
Protocol for preconditioning of the device before its characterization	[ ] Yes [x] No	No preconditioning protocol.
Stability of the J-V characteristic<br>*Verified with time evolution of the maximum power point or with the photocurrent at maximum power point; see ref. 7 for details.*	[ ] Yes [x] No	

3. Hysteresis or any other unusual behaviour

Item	Reported	Details
Description of the unusual behaviour observed during the characterization	[ ] Yes [x] No	No hysteresis was observed in our device.
Related experimental data	[ ] Yes [x] No	No.

4. Efficiency

Item	Reported	Details
External quantum efficiency (EQE) or incident photons to current efficiency (IPCE)	[x] Yes [ ] No	IPCE curve was provided in Figure 3d.
A comparison between the integrated response under the standard reference spectrum and the response measure under the simulator	[x] Yes [ ] No	Relate information is provided (5%).
For tandem solar cells, the bias illumination and bias voltage used for each subcell	[ ] Yes [x] No	Our cells were single junction solar cells.

5. Calibration

Item	Reported	Details
Light source and reference cell or sensor used for the characterization	[x] Yes [ ] No	They are provided in Method section, section of "Device Fabrication".
Confirmation that the reference cell was calibrated and certified	[x] Yes [ ] No	The reference cell was calibrated and certified.

Calculation of spectral mismatch between the reference cell and the devices under test

- [ ] Yes
- [x] No

Spectral mismatch factor was not considered.

6. Mask/aperture

Size of the mask/aperture used during testing

- [ ] Yes
- [x] No

We didn't use masks during testing in the lab.

Variation of the measured short-circuit current density with the mask/aperture area

- [ ] Yes
- [x] No

7. Performance certification

Identity of the independent certification laboratory that confirmed the photovoltaic performance

- [ ] Yes
- [x] No

Explain why this information is not reported/not relevant.

A copy of any certificate(s)
Provide in Supplementary Information

- [ ] Yes
- [x] No

Explain why this information is not reported/not relevant.

8. Statistics

Number of solar cells tested

- [x] Yes
- [ ] No

Number of solar cells tested is provided in Table 2.

Statistical analysis of the device performance

- [x] Yes
- [ ] No

Statistical results of the devices are listed in Table 2.

9. Long-term stability analysis

Type of analysis, bias conditions and environmental conditions
For instance: illumination type, temperature, atmosphere humidity, encapsulation method, preconditioning temperature

- [x] Yes
- [ ] No

Long-term stability analysis is provided in main text, section"Improved device stability
